# Advanced Image Stitching Method for Dual-Sensor Inspection

**DOI:** 10.3390/s24123778

**Published:** 2024-06-11

**Authors:** Sara Shahsavarani, Fernando Lopez, Clemente Ibarra-Castanedo, Xavier P. V. Maldague

**Affiliations:** 1 Computer Vision and Systems Laboratory (CVSL), Department of Electrical and Computer Engineering, Faculty of Science and Engineering, Laval University, Quebec City, QC G1V 0A6, Canada; xavier.maldague@gel.ulaval.ca; 2 TORNGATS, 200 Boul. du Parc-Technologique, Quebec City, QC G1P 4S3, Canada; fernando.lopez-rodriguez.1@ulaval.ca

**Keywords:** image stitching, feature matching, feature detection and description, self-supervised learning, auto-encoders, convolutional neural networks, infrared thermography, multi-modal imaging

## Abstract

Efficient image stitching plays a vital role in the Non-Destructive Evaluation (NDE) of infrastructures. An essential challenge in the NDE of infrastructures is precisely visualizing defects within large structures. The existing literature predominantly relies on high-resolution close-distance images to detect surface or subsurface defects. While the automatic detection of all defect types represents a significant advancement, understanding the location and continuity of defects is imperative. It is worth noting that some defects may be too small to capture from a considerable distance. Consequently, multiple image sequences are captured and processed using image stitching techniques. Additionally, visible and infrared data fusion strategies prove essential for acquiring comprehensive information to detect defects across vast structures. Hence, there is a need for an effective image stitching method appropriate for infrared and visible images of structures and industrial assets, facilitating enhanced visualization and automated inspection for structural maintenance. This paper proposes an advanced image stitching method appropriate for dual-sensor inspections. The proposed image stitching technique employs self-supervised feature detection to enhance the quality and quantity of feature detection. Subsequently, a graph neural network is employed for robust feature matching. Ultimately, the proposed method results in image stitching that effectively eliminates perspective distortion in both infrared and visible images, a prerequisite for subsequent multi-modal fusion strategies. Our results substantially enhance the visualization capabilities for infrastructure inspection. Comparative analysis with popular state-of-the-art methods confirms the effectiveness of the proposed approach.

## 1. Introduction

Integrating infrared (IR) and visible (VIS) imaging in industrial infrastructure inspection has proven to be a powerful approach for efficient and comprehensive assessment [1]. Both infrared and visible images offer unique insights into the condition of structures [2], enabling the detection of various defects and anomalies. However, achieving a fully automated defect detection system that identifies defects and precisely locates and assesses their continuity remains a significant challenge.

Image stitching combines multiple images with overlapping areas to create a larger panoramic image [3]. Image stitching plays an important role in enhancing the effectiveness of infrastructure inspection. Infrastructures span vast areas, and conventional imaging techniques may not capture the complete picture, especially for defects requiring more visual coverage [4]. Image stitching techniques offer a solution by seamlessly combining image sequences captured from different perspectives into a single, comprehensive representation.

Employing multi-modal images proves indispensable in achieving a comprehensive inspection and bringing together the advantages of each modality to provide a more thorough assessment of the infrastructure [5,6].

Image stitching for dual-sensor inspection takes infrastructure inspection to a higher level of accuracy and insight [7]. In different modalities, such as IR and VIS bands, the stitched images enable a more comprehensive understanding of the entire structure [4].

Automated defect detection is a crucial objective in infrastructure inspection, significantly reducing human effort and improving efficiency. The proposed approach strives to achieve automated detection across various defect types by employing multi-modal image stitching. This progress not only enhances the reliability of the inspection process but also expedites repair efforts for large structures.

This paper proposes an effective and robust method to obtain a high-precision stitched image of infrastructures to enhance defect detection. We concentrate on both improving alignment accuracy and reducing distortions. The main contributions in this work are as follows:

1. We propose a self-supervised auto-encoder feature detection technique for enhancing the quality and quantity of feature points in infrared and visible images;
2. We also employ a powerful feature matching algorithm based on graph neural networks to identify and remove the mismatched features robustly;
3. Lastly, we develop perspective-distortion-free image stitching software for dual-sensor inspection especially for low-texture conditions.

This paper is organized as follows: Section 2 presents the related works. Section 3 proposes the image stitching methodology. Then, Section 4 outlines the comparative experiments conducted on the algorithms of each part, and the final infrared and visible image stitching result is presented. Lastly, we summarize the main outcomes in Section 5.

## 2. Literature Review

### 2.1. Feature Detection and Descriptor

Learning-based feature detection methods are divided into supervised [8], self-supervised [9,10], and unsupervised [11,12,13,14,15]. These methods are often reformulated as regression problems, enabling trainable models that remain robust to various transformations and imaging conditions. However, the effectiveness of supervised methods heavily relies on the construction of anchors, which often poses limitations and prevents the accurate proposal of keypoints [15].

Many methods integrate feature detection into the matching pipeline, enhancing overall performance and optimizing the procedure end to end. TILDE [16] trains regression models for repeatable keypoints under diverse imaging conditions. DetNet [11] formulates learning local covariant features as a regression problem with covariance constraints. Quad-net [12] achieves keypoint detection under transformation-invariant quantile ranking.

### 2.2. Feature Matching

An overview of image matching methods, categorizing them into area-based and feature-based methods, is presented in [17,18]. Area-based methods operate without feature detection, while feature-based methods involve extracting feature points and descriptors, with direct and indirect matching approaches.

Direct feature matching establishes correspondences using spatial geometrical relations, including graph matching and point set registration. Indirect feature matching involves a two-stage process, starting with preliminary correspondences and applying geometrical constraints to remove false matches. Dense matching requires post-processing for transform model estimation and image resampling.

Learning-based methods, introduced separately, use images and point data for improved performance. Correlation-like methods maximize similarities of sliding windows, while domain-transformed methods align images by converting them into another domain. Mutual information (MI) methods measure statistical dependency between images, suitable for multi-modalities. MI, despite its utility, faces challenges in determining the global maximum. Different optimization methods and transformation models are discussed.

The area-based methods are suitable for specific applications like medical or remote sensing image registration, where feature-based methods may struggle. However, area-based methods face challenges with geometrical transformations and local deformations. Research works presented in [17,19,20,21] hint at the integration of deep learning into area-based matching for improved efficacy, a topic to be reviewed in the learning-based matching section. Graph matching (GM) involves associating feature points to nodes, forming a graph for investigating the image data structure. GM addresses the establishment of node-to-node correspondences between graphs, classified as exact and inexact matching. While exact matching is too strict, researchers often opt for inexact matching with weighted attributes on nodes and edges. The focus of this survey is on inexact matching methods. GM formulates the feature matching problem, encoding geometrical cues into node and edge affinities. The recent GM form is a Quadratic Assignment Problem (QAP), with Lawler’s QAP being a primary focus. Koopmans–Beckmann’s QAP is another formulation, related to Lawler’s. GM aims to find optimal one-to-one correspondences, facing NP-hardness. Researchers in [22,23,24] used various relaxation strategies, and GM solvers are introduced in the literature.

#### Image Stitching

Image stitching or image mosaicking involves obtaining a wider field of view of a scene from a sequence of partial views [25]. Image stitching deals with low overlapping images and requires accurate alignment at the pixel level to avoid visual discontinuities. Feature-based stitching methods are popular because of their invariance properties and efficiency. For example, in order to identify geometrically consistent feature matches and achieve accurate homography estimation, Brown and Low [26] proposed using the SIFT [27] feature matching and the RANSAC [28] algorithm. Lin et al. [29] also used SIFT to precompute matches and then jointly estimate the matching and the smoothly varying affine fields for better stitching performance.

## 3. Materials and Methods

**Figure 1:**
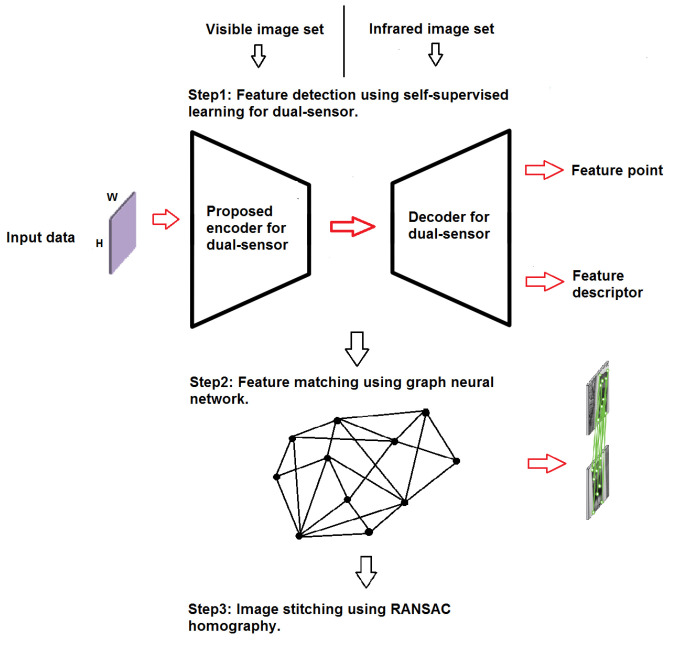
Advanced image stitching method for dual-sensor inspection. (General scheme of the proposed method [4]).

This section introduces the proposed image stitching methodology. Figure 1 indicates the general scheme of the method. This method includes three phases. Each phase will be explained in the following subsections. Also, Algorithm 1 shows the implementation of the proposed method.
**Algorithm 1: Advance Image Stitching Algorithm for Dual-Sensor Inspection**
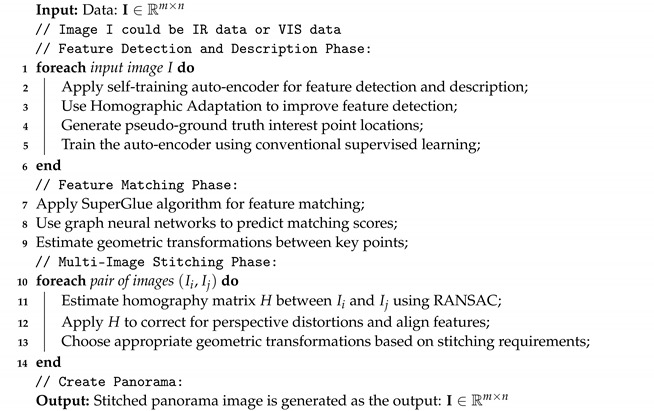


### 3.1. Feature Detection and Description Phase

This subsection presents a feature detection and description methodology specifically designed for addressing complexities arising in poor or low-texture conditions, which are particularly prevalent in infrared as well as visible images related to certain materials such as concrete. The challenges associated with such conditions include both the scarcity and suboptimal quality of feature points [30,31]. Consequently, we propose a method aimed at enhancing both the quantity and quality of feature points. The primary objective is to address issues such as the lack of repetition of feature points in overlapping areas, homogeneity, and the sparse distribution of feature points.

#### Self-Training Auto-Encoder for Unified Dual-Sensor Feature Detection and Description

To this end, we employ an auto-encoder for feature detection and description through a self-supervised approach. Auto-encoders prove to be powerful feature learning algorithms capable of automatically discovering and representing complex patterns and hierarchical features in the data [32].

**Figure 2:**
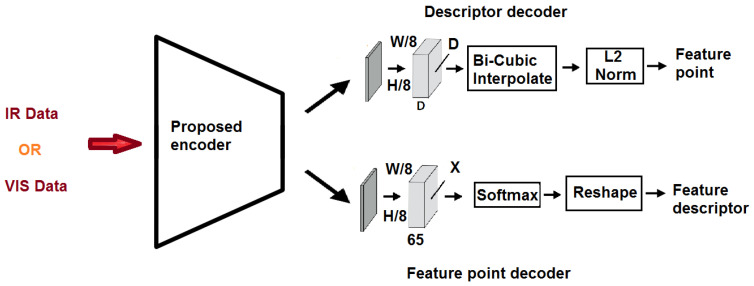
The proposed auto-encoder for feature detection and description.

**Figure 3:**
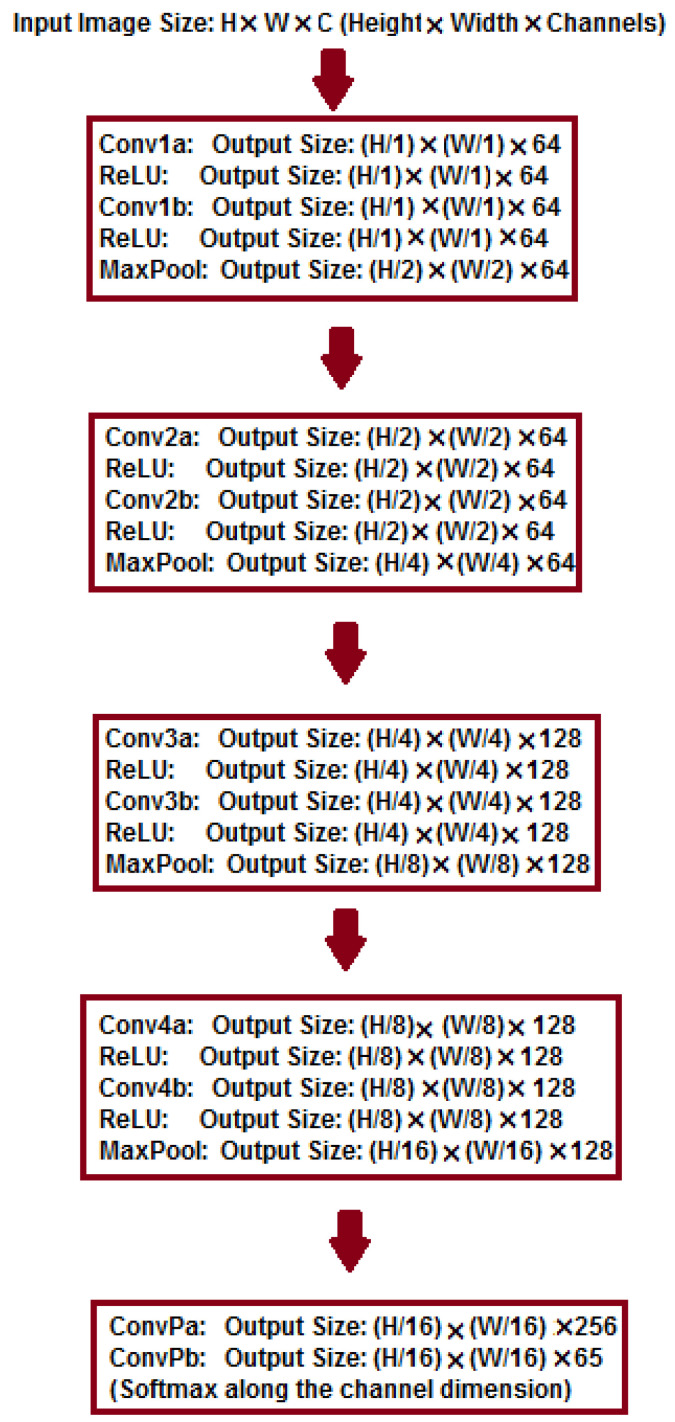
The proposed fully convolutional neural network as the shared encoder (refer to Figure 2).

Figure 2 illustrates the proposed feature detection and description method, inspired by [10], which employs an auto-encoder to enhance the efficiency of feature point detection and description. Auto-encoders consist of two primary components: the encoder and decoder [32,33]. A VGG-like [34] convolutional neural network is designed and implemented as the shared encoder. Figure 3 shows the fully convolutional neural network with all the details as the shared encoder. We mitigate inaccurate feature detection by exploiting the inherent dimensionality reduction advantage of encoders.

The proposed network is designed to perform effectively across diverse image modalities. As Figure 2 illustrates, the network maps the input image $I\in R^{H\times W}$ to an intermediate tensor $B\in R^{H_{c}\times W_{c}\times F}$. This computation involves two headers, a 2D interest point detector head, and a descriptor head. The 2D detector head computes $X\in R^{H_{c}\times W_{c}\times 65}$. Following channel-wise softmax [35] and non-maximal suppression (NMS) [36], the detector organizes the detected feature points based on provided confidence levels and selects *k* feature points with the highest confidence as the output. To reduce computation and memory usage, the descriptor learns semi-dense descriptors $D\in R^{H_{c}\times W_{c}\times D}$. Subsequently, the bicubic interpolation algorithm is applied to obtain complete descriptors of size $R^{H_{c}\times W_{c}\times D}$, and, finally, L2 normalization is employed to obtain unit length descriptions.

Within the auto-encoder, a self-supervised framework plays an important role. The self-supervised network’s architecture is meticulous. Homography adaptation is employed for applying various homographies to a single image, representing distinct views of the same data [10,37]. Feature detection and description are initially applied to each view, culminating in a feature fusion step. This approach enhances the detection of a maximal quantity of feature points. The proposed method initiates its functionality using a foundational interest point detector and an extensive collection of unlabeled images from the target domain, such as MS-COCO [38]. Employing a self-supervised paradigm, also recognized as self-training, we initially generate a set of pseudo-ground truth interest point locations for each image in the target domain. Subsequently, we utilize conventional supervised learning techniques.

Central to the proposed approach is a procedure that involves applying random homographies to warped duplicates of the input image and merging the outcomes—a technique we refer to as Homographic Adaptation. Homographies provide precise or nearly precise transformations between images, particularly suited for camera motion characterized by rotation around the camera center. Additionally, given that a significant portion of the world exhibits reasonably planar characteristics, a homography is an effective model for representing the changes observed when the same 3D point is viewed from different perspectives. An advantage is that homographies do not necessitate 3D information, allowing for random sampling and straightforward application to any 2D image with minimal computational overhead, typically involving bilinear interpolation. Due to these advantages, homographies constitute the foundational element of the proposed self-supervised approach. Let $f_{\theta }\left(\cdot \right)$ denote the initial interest point function we aim to adapt, *I* the input image, *x* the resulting interest points, and *H* a random homography such that

(1) $$x=f_{\theta }\left(I\right)$$

An ideal interest point operator should be covariant with respect to homographies. A function $f_{\theta }\left(\cdot \right)$ is covariant with *H* if the output transforms with the input. In other words, a covariant detector will satisfy, for all $H\in R^{3}$,

(2) $$Hx=f_{\theta }\left(H\left(I\right)\right)$$

Moving homography-related terms to the right, we obtain

(3) $$x=H^{-1}f_{\theta }\left(H\left(I\right)\right)$$

In practice, a detector will not be perfectly covariant—different homographies in Equation (3) will result in different interest points *x*. The basic idea behind Homographic Adaptation is to perform an empirical sum over a sufficiently large sample of random *H* values. The resulting aggregation over samples thus gives rise to a new and improved super-point detector, $\hat{F}\left(\cdot \right)$:

(4) $$\hat{F}\left(I;f_{\theta }\right)=\frac{1}{N_{h}}\sum_{i=1}^{N_{h}}H_{i}^{-1}f_{\theta }\left(H_{i}\left(I\right)\right)$$

### 3.2. Feature Matching Phase

SuperGlue [39] is a graph neural-network-based algorithm designed for feature matching in computer vision tasks, particularly for establishing correspondences between key image points. Developed for tasks such as image matching, stereo matching, and visual localization, SuperGlue raises deep neural networks to predict matching scores and estimate the geometric transformation between keypoints.

Geometric transformations can be applied during image stitching to correct for perspective distortions and improve the alignment of images in a mosaic or panorama. The SuperGlue algorithm potentially has this opportunity. The goal is to create a visually seamless and undistorted composite image by compensating for variations in viewpoint, rotation, and scale across the individual images. In the context of image stitching, common geometric transformations include the following:

- Translation: Shifting the position of one image relative to another;
- Rotation: Rotating an image to align features;
- Scaling: Adjusting the size of an image to match the scale of the reference image;
- Homography: A more general transformation that includes translation, rotation, scaling, and skewing. It is particularly useful for correcting perspective distortions.

By applying these transformations to each image before stitching, the software aims to minimize perspective distortions and achieve a smooth transition between adjacent images. This process is crucial, especially when images are captured from different viewpoints or with variations in camera parameters. In the following, it is explained how geometric transformations contribute to distortion correction:

- Perspective Distortion Correction: Geometric transformations, such as homography, can correct for perspective distortions when objects in the scene are viewed from different angles. It is particularly relevant when capturing images with wide-angle lenses or from non-ideal shooting positions;
- Seamless Alignment: Applying appropriate transformations ensures that key features in the overlapping regions of adjacent images align correctly. This alignment is critical for creating a visually coherent and distortion-free stitched image;
- Global Adjustment: Geometric transformations allow for global adjustments, ensuring that the entire set of images contributes cohesively to the stitched result. This is essential for avoiding artifacts and maintaining a natural appearance.

### 3.3. Multi-Image Stitching Phase

The primary goal of this section is to create a panorama or mosaic that seamlessly integrates the input images. One crucial step in this process is estimating the homography between pairs of images, and the Random Sample Consensus (RANSAC) [28] algorithm is often used for robust homography estimation.

The homography matrix (H) is a 3×3 transformation matrix that relates points in one image to their corresponding points in another image:

$$\left[\begin{array}{c} x^{\prime }\\ y^{\prime }\\ 1 \end{array}\right]=H\left[\begin{array}{c} x\\ y\\ 1 \end{array}\right]$$

where (*x*, *y*) are the coordinates in one image, and ($x^{\prime }$, $y^{\prime }$) are the coordinates in the other image.

The RANSAC algorithm is beneficial when dealing with scenarios with outliers, noise, or incorrect correspondences in the matching process. Hence, the probably false correspondences will also be eliminated in this section. It provides a robust way to estimate transformations like homographies in the presence of such challenges.

## 4. Results and Discussion

In this section, we investigate a comprehensive analysis of the results obtained in this study, shedding light on the essential findings and their implications. This research focused on image stitching using dual-sensor infrared and visible spectra to enhance Non-Destructive Evaluation inspection. Throughout the experiments, we examined various factors and variables. This section provides a structured overview of these results, beginning with the detailed feature detection and description phase, then the in-depth discussion of feature point matching, and concluding with insights into image stitching. By systematically dissecting our findings, we aim to uncover patterns, draw meaningful conclusions, and offer insights into the broader implications of this research, contributing to a deeper understanding of image stitching using multi-sensors for infrastructure visualization.

### 4.1. Dataset and Implementation Details

This study uses two datasets containing coupled thermal and visible images of industrial assets to evaluate the proposed approach. The first dataset is related to a wall of concrete. A Zenmuse H20T camera (Da-Jiang Innovations Science and Technology Co., Ltd., Shenzhen, China) collected coupled thermal and visible images with 640×512 resolution. This dataset contains 86 coupled IR and thermal images. The other dataset is related to the roof of a building. This dataset includes 73 coupled thermal and visible images. A DJI M300 drone (Da-Jiang Innovations Science and Technology Co., Ltd., Shenzhen, China) equipped with a Zenmuse H20T camera was employed for acquiring thermal and visible images. The images of both datasets have almost 75% overlap. Both dataset are captured at an altitude of 15 m. All the experiments in this paper were implemented in Python programming language on a computer with a 2080Ti GPU.

### 4.2. Results of Feature Detection and Description Phase

In this subsection, we compare several popular feature detection and description algorithms to verify the applicability of the proposed feature detection and description method in complex conditions such as those with poor or low texture conditions, repetitive patterns, and homogeneity, which mostly take place in IR thermography images as well as visible images of some materials like concrete.

**Figure 4:**
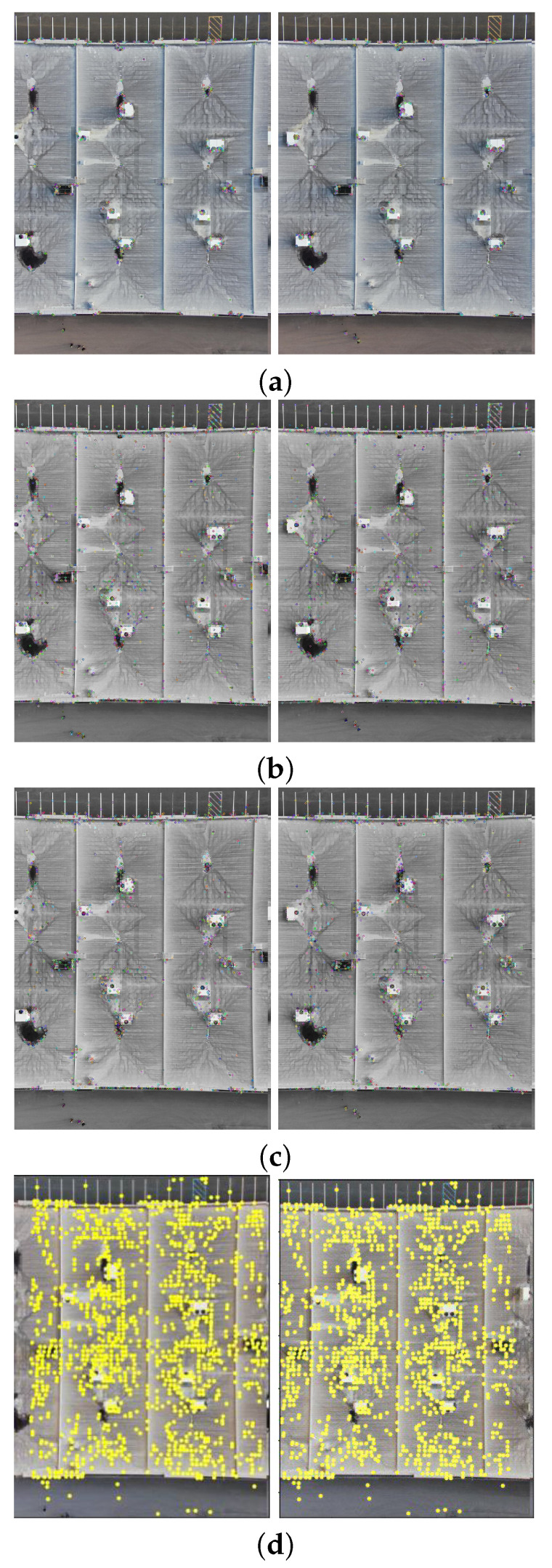
Feature detector performance results on visible image dataset 1 for two consecutive images. (**a**) ORB features are dense in boundaries and edges. (**b**) SIFT, (**c**) AKAZE, (**d**) the proposed method. Challenges and complexities: repetitive patterns on the surface, homogeneity, and poor texture in some parts on the surface.

**Figure 5:**
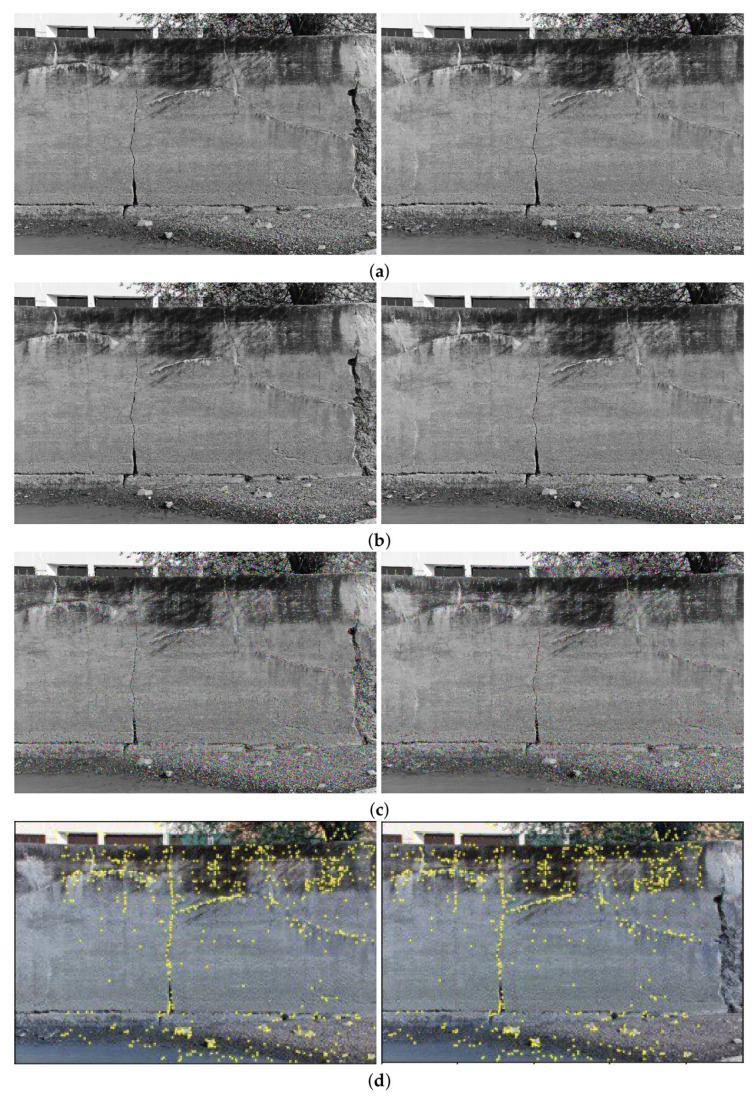
Feature detector performance results on visible image dataset 2 for two consecutive images. (**a**) ORB features, (**b**) SIFT features, (**c**) AKAZE features, (**d**) the proposed method. Challenges and complexities: repetitive patterns on the surface, homogeneity, lack of texture.

**Figure 6:**
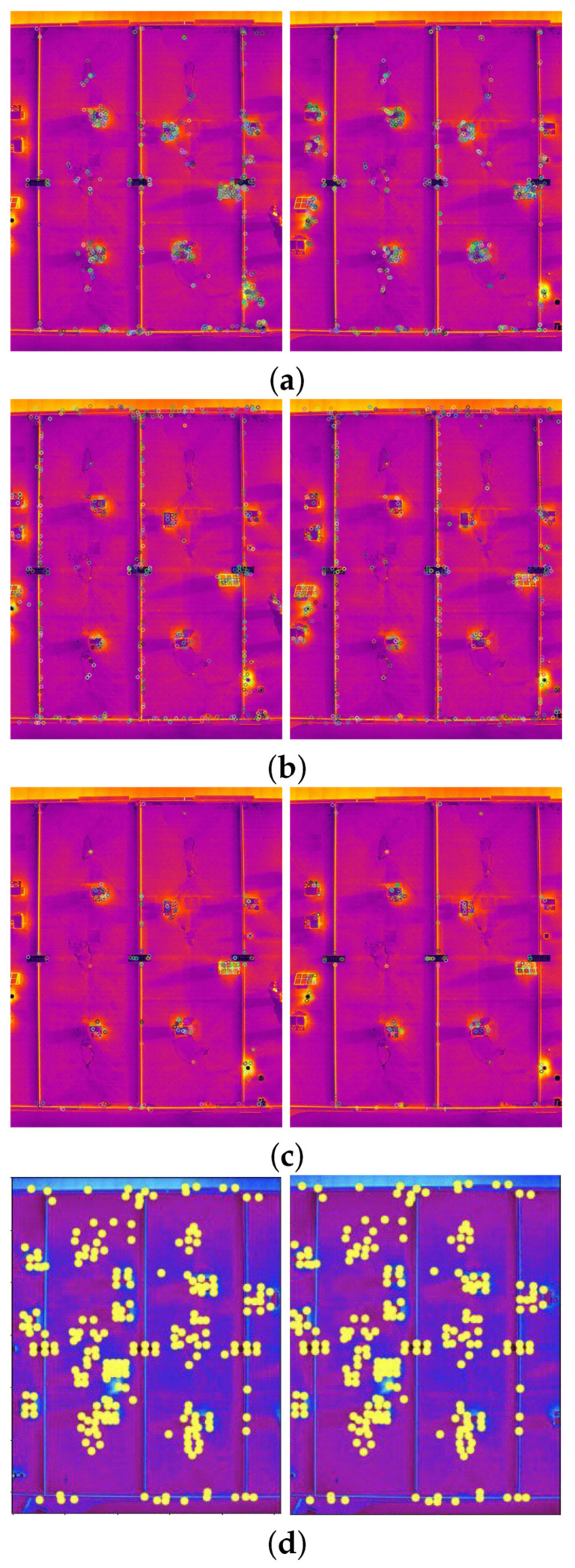
Feature detector performance results on infrared image dataset 1 for two consecutive images. (**a**) ORB features, (**b**) SIFT, (**c**) AKAZE, (**d**) the proposed method feature points. Challenges and complexities: repetitive patterns on the surface, homogeneity, and lack of texture.

**Figure 7:**
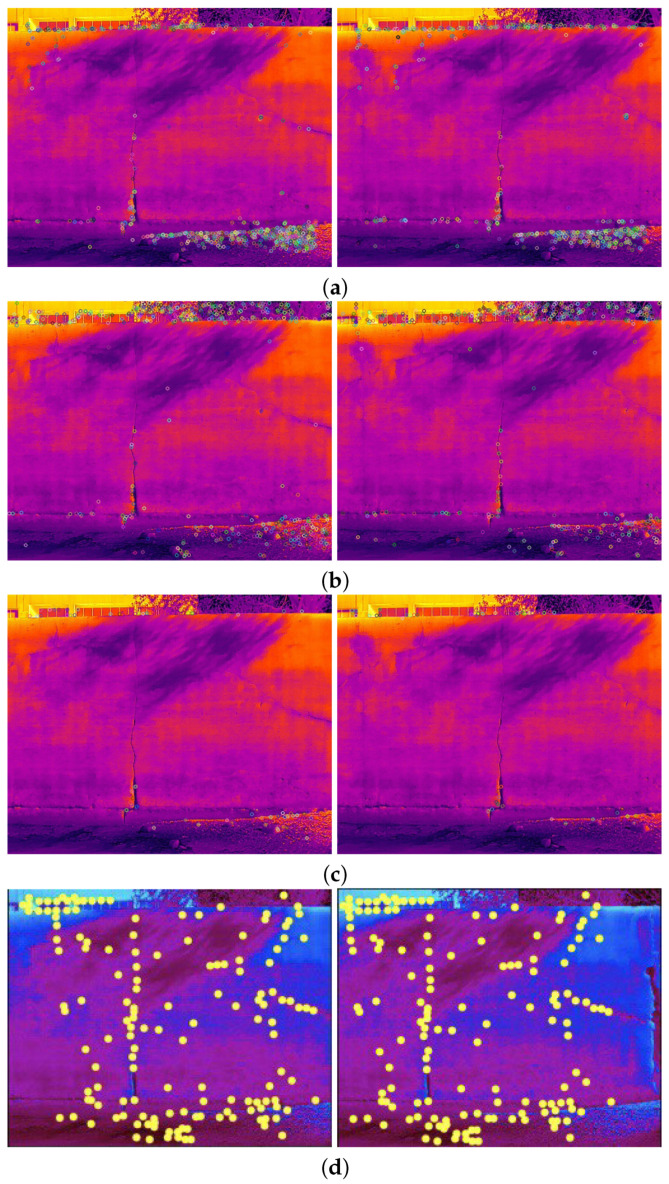
Feature detector performance results on infrared image dataset 2 for two consecutive images. (**a**) ORB features, (**b**) SIFT features, (**c**) AKAZE features, (**d**) the proposed method feature points. Challenges and complexities: repetitive patterns on the surface, homogeneity, and lack of texture.

The comparison methods include ORB [40], AKAZE [41], and SIFT, which are highly trusted methods which have been used for decades by researchers for feature detection and description. We applied these methods to visible and infrared images, then compared them with proposed method. We tested 160 IR images and 160 visible images of the industrial structures we mentioned earlier. There is a significant difference in the feature point’s quantity and quality between the proposed methods and other methods. Also, the repeatability of the detected feature in the sequence images is dramatically visible, especially with low or poor textures and repetitive patterns, as shown in the results of the test images in Figure 4, Figure 5, Figure 6 and Figure 7.

Poor Texture: Structures like concrete often have large, monotonous, and texture-less surfaces. Traditional feature detectors like ORB, SIFT, and AKAZE rely on identifying distinctive texture patterns. As shown in Figure 4, Figure 5, Figure 6 and Figure 7, as the texture becomes less and less defined, they struggle to find suitable feature points, resulting in a lack of feature matches. The proposed method finds the critical points more precisely.

Reflectance Variations: Complex structures can exhibit varying degrees of reflectance under different lighting conditions. Feature detectors are sensitive to illumination changes and might produce inconsistent results when lighting conditions fluctuate. This can lead to unreliable feature points.

Parallax: Parallax occurs when the viewpoint changes, causing objects to appear at different positions in images taken from different angles. Traditional feature detectors, designed for planar scenes, may not handle parallax well. They might generate feature points that do not align correctly between images with significant viewpoint variations.

Homogeneity: IR and VIS structures often have large, uniform regions where pixel values do not vary significantly. Traditional detectors like ORB, SIFT, and KAZE struggle to identify feature points in such homogeneous areas, leading to sparse feature point distributions.

Repetition pattern: Repetitive patterns, common in concrete structures, can confuse feature detectors like ORB. These detectors may produce numerous feature points on repeated patterns, making it challenging to match them accurately.

In Figure 4, we present a visual representation of two successive images in each row exhibiting a 0.75 overlap. In the case illustrated in Figure 4a, we focus on visible images characterized by repetitive patterns and homogeneity. The ORB feature detector is employed in this scenario, with its primary strength lying in the identification of points along edges. However, it is noteworthy that the number of points identified by ORB is somewhat constrained. Moreover, we anticipate the need for more repetition of feature points in areas where the two sequence images overlap.

This observation leads us to Figure 4b,c, where we explore using SIFT and AKAZE feature detectors. These detectors prove more adept at identifying feature points compared to ORB. However, challenges persist regarding repeating these detected points in the overlapped areas.

In the pursuit of addressing these challenges, Figure 4d shows the proposed method, which enhances detecting feature points. Notably, the proposed method demonstrates a substantial increase in the number of detected feature points, including those repetitively identified in the overlapped regions. For a more rigorous assessment of the proposed method’s efficacy, shown in Figure 5, we examine a scenario posing increased difficulty. Here, the concrete surface presents a formidable challenge for SIFT and ORB, which struggle to detect feature points effectively. In contrast, the proposed method significantly outperforms these traditional detectors, showcasing a superior ability to detect a more substantial number of feature points, particularly in regions where image overlap occurs. It emphasizes the robustness and effectiveness of the proposed method, especially in challenging environmental conditions.

### 4.3. Results of Feature Matching Phase

**Figure 8:**
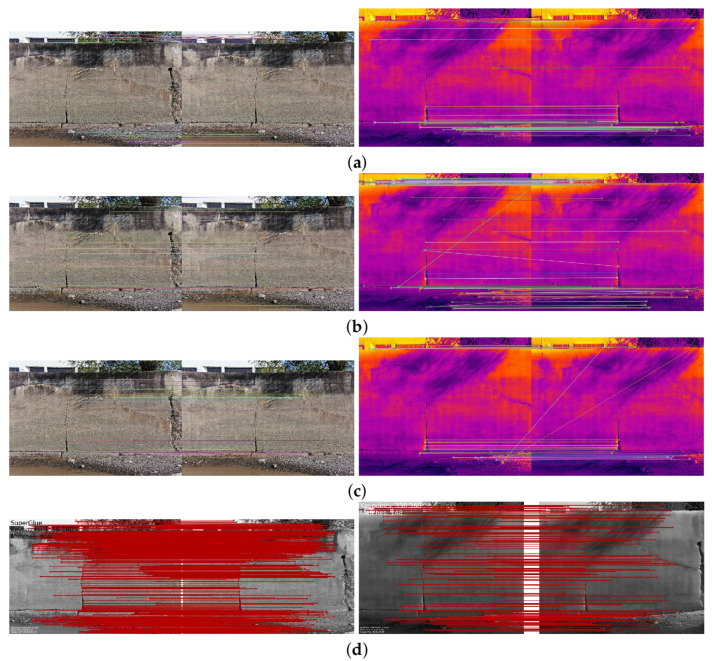
Feature matching performance for two consecutive visible images and their counterpart infrared images in dataset 2. (**a**) ORB + BFMatcher, (**b**) SIFT + BFMatcher, (**c**) AKAZE + BFMatcher, (**d**) proposed method. Explanation: feature matching performance through proposed method is dramatically promising.

**Figure 9:**
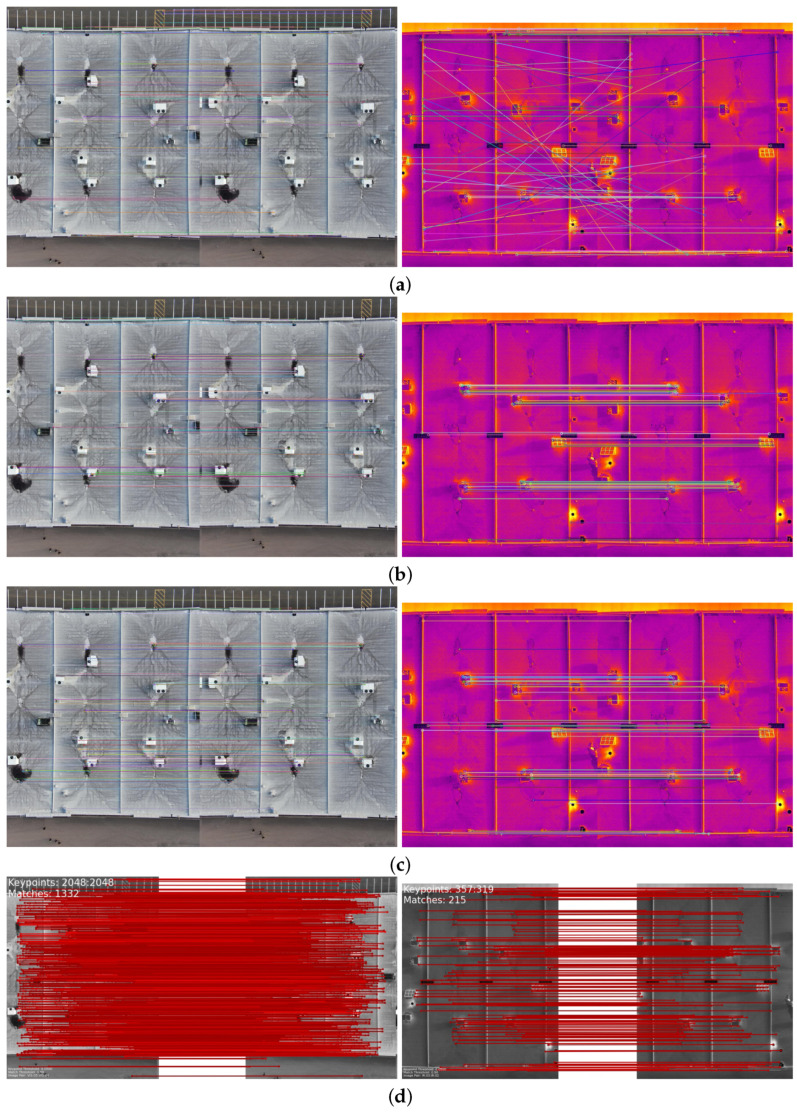
Feature matching performance for two consecutive visible images and their counterpart infrared images. (**a**) ORB + BFMatcher, (**b**) SIFT + BFMatcher, (**c**) AKAZE + BFMatcher, (**d**) proposed method. **Explanations:** Feature matching performance through proposed method is dramatically promising. There is some false feature matching in (**a**) ORB + BFMatcher for infrared images.

As Figure 8 and Figure 9 indicate, ORB, SIFT, and AKAZE methods usually perform well for scenarios with richer textures, consistent lighting, and minimal parallax. These methods yield suboptimal results when applied to structures with poor texture, reflectance variations, parallax effects, homogeneity, and repetition. This can lead to the following:

- Inaccurate matches: Keypoints may not align correctly between images due to parallax or poor texture, leading to incorrect feature matches such as those shown Figure 8a–c and Figure 9a;
- Difficulty in handling repetitive patterns: Traditional methods may struggle to distinguish between repeated patterns, leading to ambiguous matches;
- Repeatability: Changes in lighting conditions and surface reflectance can result in inconsistent feature points and matches;
- The proposed method specializes in feature detection and description methods appropriate for complex structures which can adapt to these unique conditions and provide more reliable infrastructure inspection and assessment results.

In our comprehensive comparison of feature point matching methods for visible and infrared images, an in-depth analysis reveals nuanced differences in performance across various scenarios. While ORB, SIFT, and AKAZE methods demonstrate proficiency in environments characterized by richer textures, consistent lighting, and minimal parallax, their efficacy diminishes when confronted with challenges such as poor texture, reflectance variations, parallax effects, homogeneity, and repetition within structures.

Remarkably, the feature matching results of ORB, especially when applied to infrared images, exhibit a notable degree of error, contributing to suboptimal outcomes. Furthermore, the total number of feature matches achieved by ORB, SIFT, and AKAZE is consistently eclipsed by the proposed method, underscoring its superiority in addressing the challenges posed by complex structural environments.

This discrepancy in performance can be attributed to several factors:

1. Sparse Keypoint Distributions: The analysis of ORB and SIFT feature points reveals a sparse distribution, limiting the possibilities for feature matching. This scarcity can hinder the overall effectiveness of these methods;
2. Inaccurate Matches: The inherent limitations of ORB, SIFT, and AKAZE become apparent in scenarios involving parallax or poor texture, where keypoints may fail to align accurately between images. This discrepancy results in incorrect feature matches, adversely affecting the reliability of the matching process;
3. Difficulty in Handling Repetitive Patterns: Traditional methods, including ORB and SIFT, face challenges in distinguishing between repeated patterns. This difficulty leads to ambiguous matches, introducing uncertainty into the feature matching outcomes;
4. Repeatability Challenges: Changes in lighting conditions and surface reflectance pose challenges to the repeatability of feature points and matches in ORB, SIFT, and AKAZE. This inconsistency in performance can compromise the reliability of these methods in real-world applications.

In response to these limitations, the proposed method is designed to specialize in feature detection and description appropriate to the complex conditions of infrastructures. This adaptability enhances the reliability of infrastructure inspection and assessment results, making it a more robust choice for scenarios where traditional methods fall short. The proposed method stands out as an innovative approach, offering improved feature matching outcomes and demonstrating a capacity to handle the intricacies of diverse and challenging structural environments.

### 4.4. Results of Image Stitching Phase

This subsection provides the final results of this research, which navigates through diverse scenarios extracted from two distinct datasets.

In the intricate process of multi-image stitching, the proposed method enhances the homography estimation between each image sequence through improving the quality and quantity of feature points. This computation draws upon mathematical formulations shaped by the feature points identified in Phase 1 and subsequently validated in Phase 2, the image matching phase. This study illuminates the important role played by both the quality and quantity of feature points in influencing the efficacy of the stitching process.

Moreover, as part of our strategy to enhance mosaic creation, we integrate the SuperGlue algorithm. This algorithm efficiently identifies and eliminates mismatched points and outliers, generating a significantly more accurate transformation between feature points in consecutive images at each stitching step. The outcome is the creation of image mosaics characterized by heightened quality, minimized distortion, and a more promising perspective.

This innovative approach bears potential for not only industrial infrastructure inspection but also for a spectrum of applications, especially those requiring distortion-free and high-quality image mosaicking, such as remote sensing, and visual documentation for heritage preservation. The integration of mathematical precision and algorithmic sophistication propels the proposed methodology to the forefront of advancements in image stitching technology.

In the following pages, the final results of the proposed image stitching method using the dual-sensor infrared images and visible images of two different datasets with different scenarios are illustrated. In the next pages, eighteen different scenarios are tested. In particular, the image stitching method is tested with nine scenarios of visible image stitching, and nine scenarios of infrared image stitching are tested. Therefore, the proposed method is tested using different numbers of data from seven to seventeen in each test.

**Figure 10:**
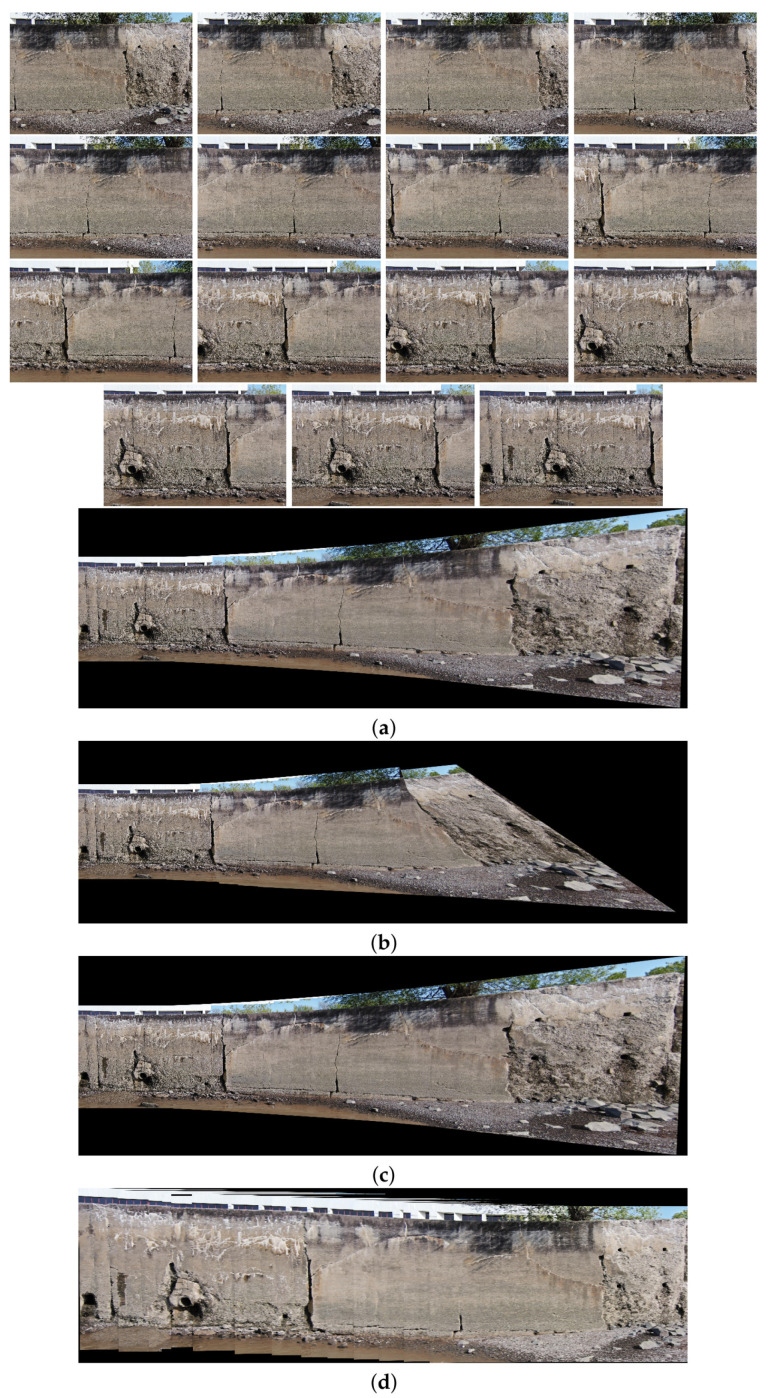
Image stitching for fifteen visible images of dataset 2. (**a**) AKAZE + BFMatcher: the stitched image has a high perspective distortion; (**b**) ORB + BFMatcher: the stitched image has less perspective distortion and shape deformation; (**c**) SIFT + BFMatcher: the stitched image has a high perspective distortion; (**d**) proposed method: the stitch image is regular and perspective distortion free.

**Figure 11:**
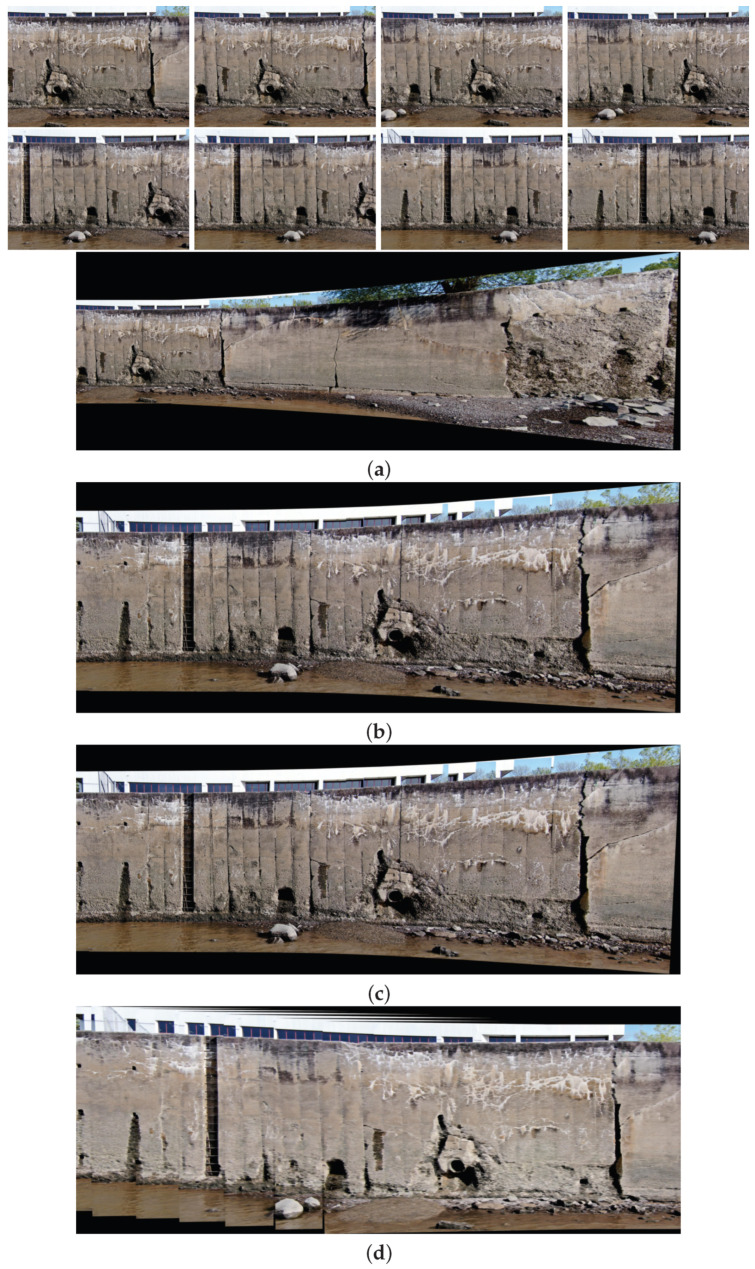
Image stitching for eight visible images of dataset 2. (**a**) AKAZE + BFMatcher: the stitched image has a high perspective distortion; (**b**) ORB + BFMatcher: the stitched image has less perspective distortion and shape deformation; (**c**) SIFT + BFMatcher: the stitched image has a high perspective distortion; (**d**) proposed method: the stitch image is regular and perspective distortion free.

**Figure 12:**
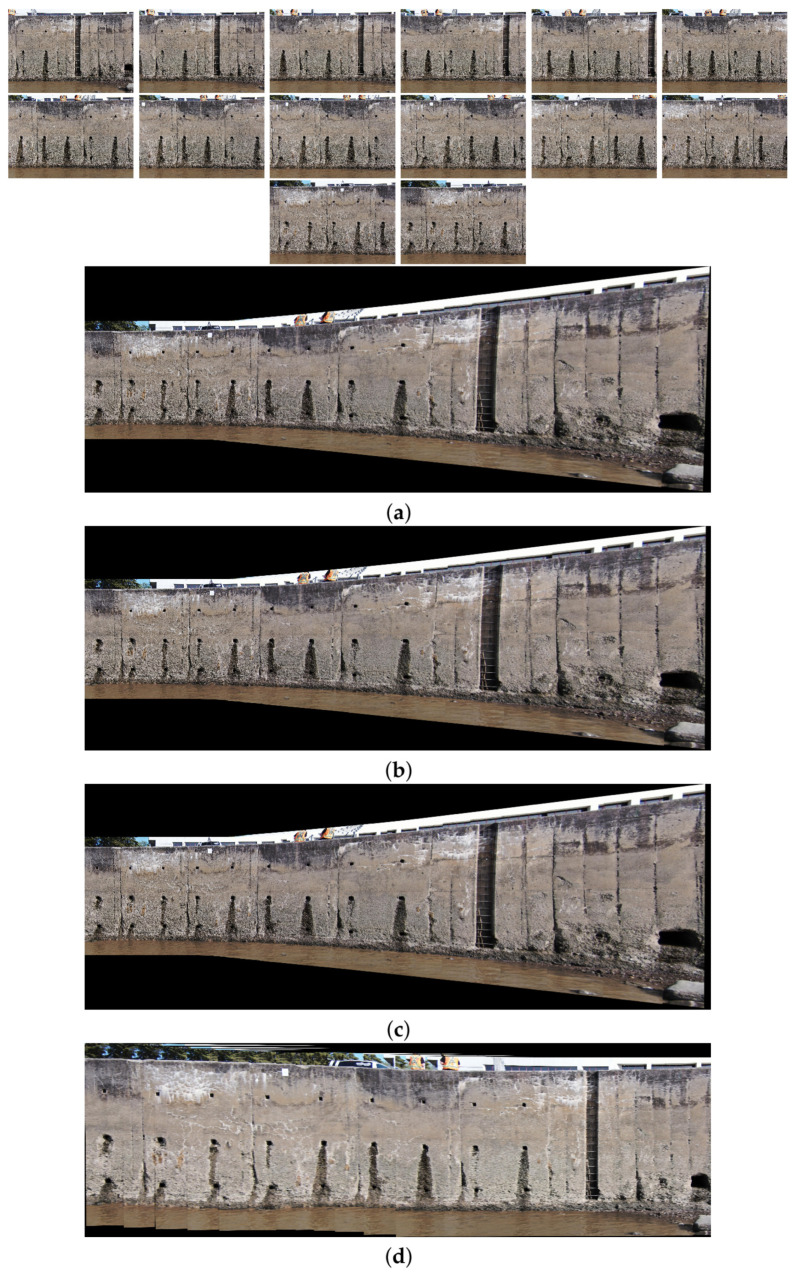
Image stitching for fourteen visible images of dataset 2. (**a**) AKAZE + BFMatcher: the stitched image has a high perspective distortion; (**b**) ORB + BFMatcher: the stitched image has a high perspective distortion; (**c**) SIFT + BFMatcher: the stitched image has a high perspective distortion; (**d**) proposed method: the stitch image is regular and perspective distortion free.

**Figure 13:**
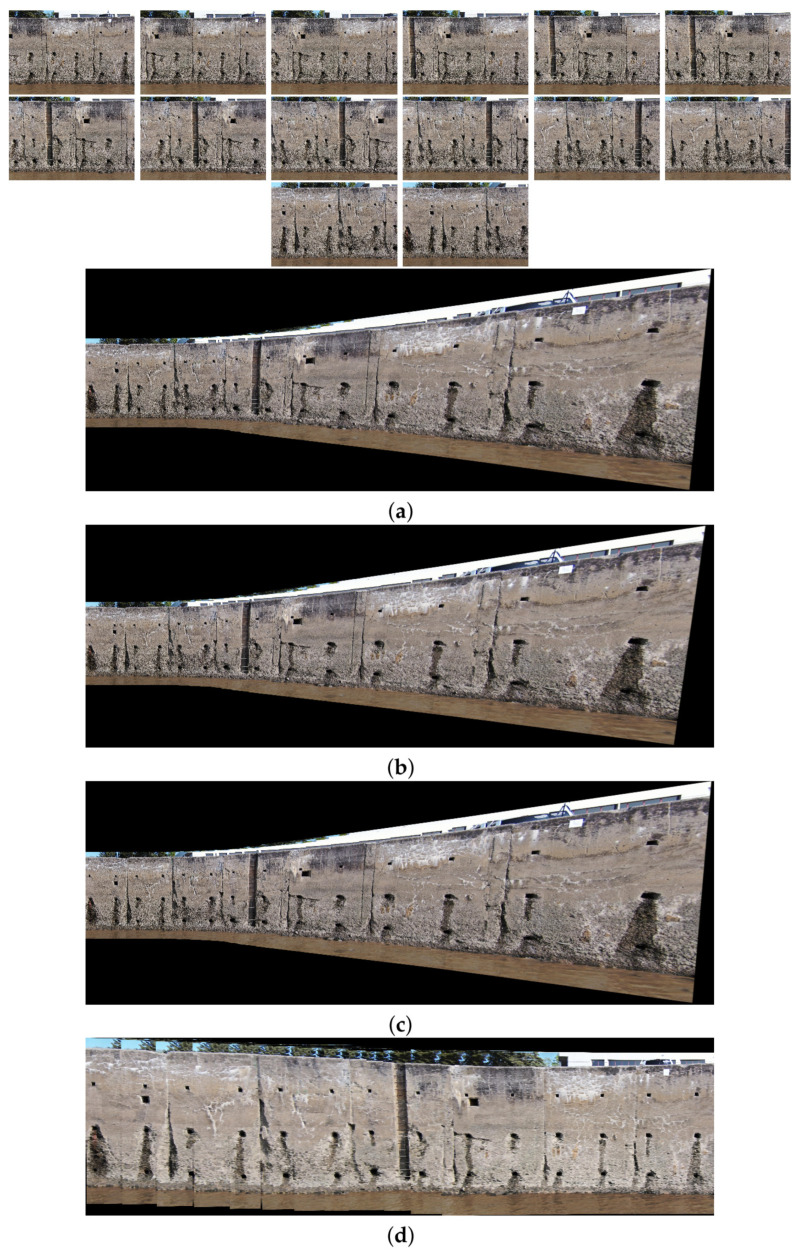
Image stitching for fourteen visible images of dataset 2. (**a**) AKAZE + BFMatcher: the stitched image has a high perspective distortion; (**b**) ORB + BFMatcher: the stitched image has a high perspective distortion; (**c**) SIFT + BFMatcher: the stitched image has a high perspective distortion; (**d**) proposed method: the stitch image is regular and perspective distortion free.

**Figure 14:**
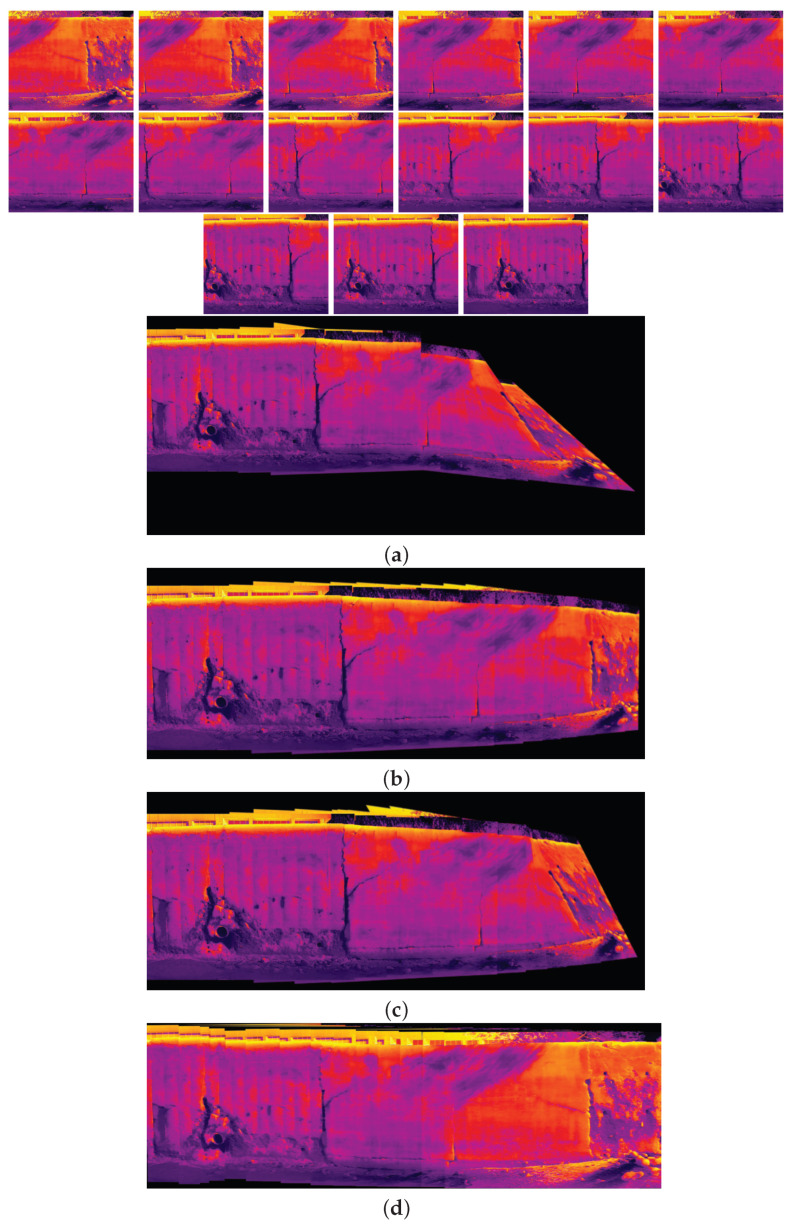
Image stitching for fifteen images of dataset 2. (**a**) AKAZE + BFMatcher: the stitched image has a shape deformation and high perspective distortion; (**b**) ORB + BFMatcher: the stitched image has a perspective distortion; (**c**) SIFT + BFMatcher: the stitched image has a perspective distortion; (**d**) proposed method: the stitch image is regular and perspective distortion free.

**Figure 15:**
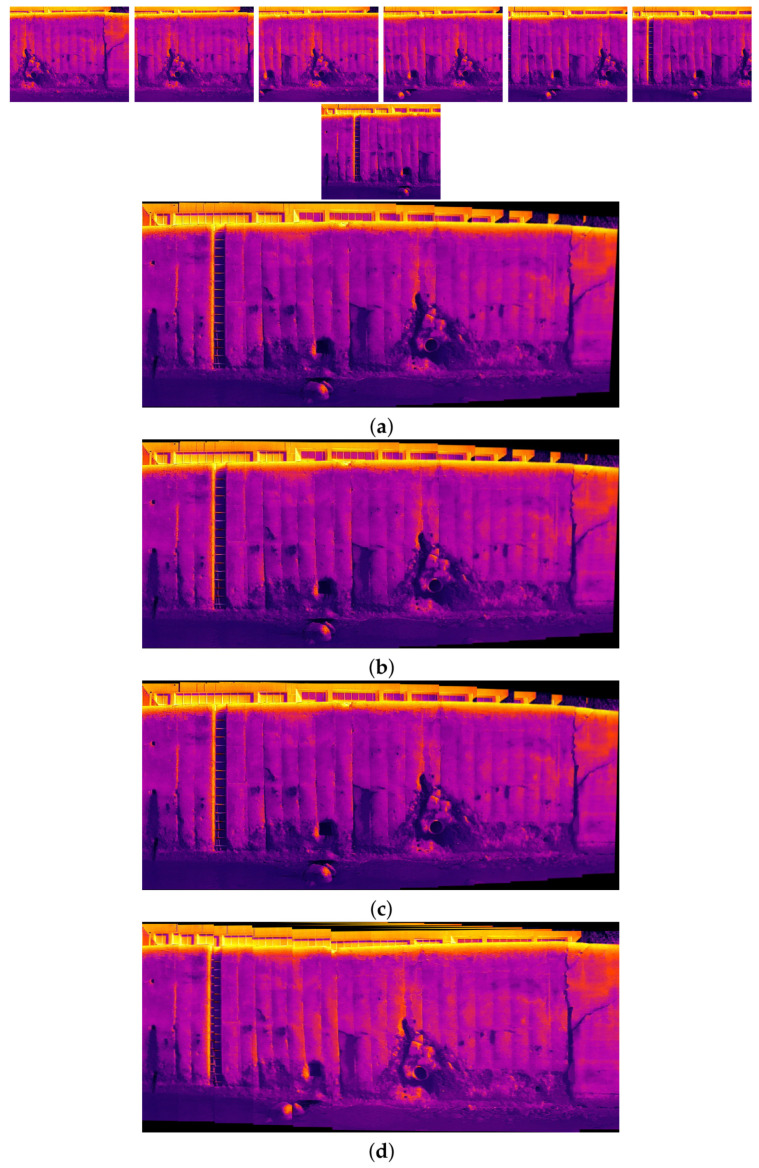
Image stitching for seven images of dataset 2. (**a**) AKAZE + BFMatcher: the stitched image has perspective distortion; (**b**) ORB + BFMatcher: the stitched image has perspective distortion; (**c**) SIFT + BFMatcher: the stitched image has perspective distortion; (**d**) proposed method: the stitched image is regular and perspective distortion free.

**Figure 16:**
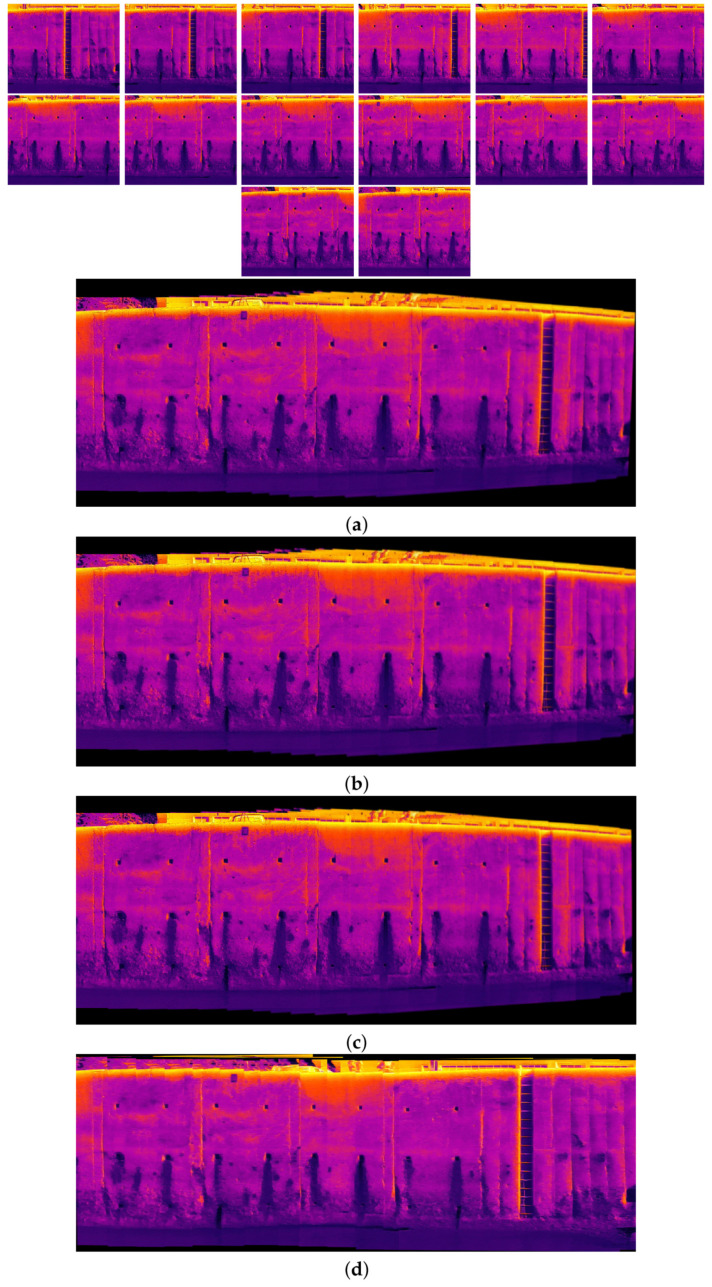
Image stitching for fourteen images of dataset 2. (**a**) AKAZE + BFMatcher: the stitched image has perspective distortion; (**b**) ORB + BFMatcher: the stitched image has perspective distortion; (**c**) SIFT + BFMatcher: the stitched image has perspective distortion; (**d**) proposed method: the stitched image is regular and perspective distortion free.

Figure 10, Figure 11, Figure 12 and Figure 13 illustrate four scenarios of the visible images of dataset 2 and the final results of the proposed image stitching method. As the figures indicate, the proposed method brings a panorama perspective-distortion-free image in comparison to the other methods. Figure 14, Figure 15 and Figure 16 indicate three scenarios of the counterpart infrared images and the final results of the proposed image stitching method. As the figures indicate, the proposed method brings out a panorama-perspective distortion-free image in comparison to the other methods.

**Figure 17:**
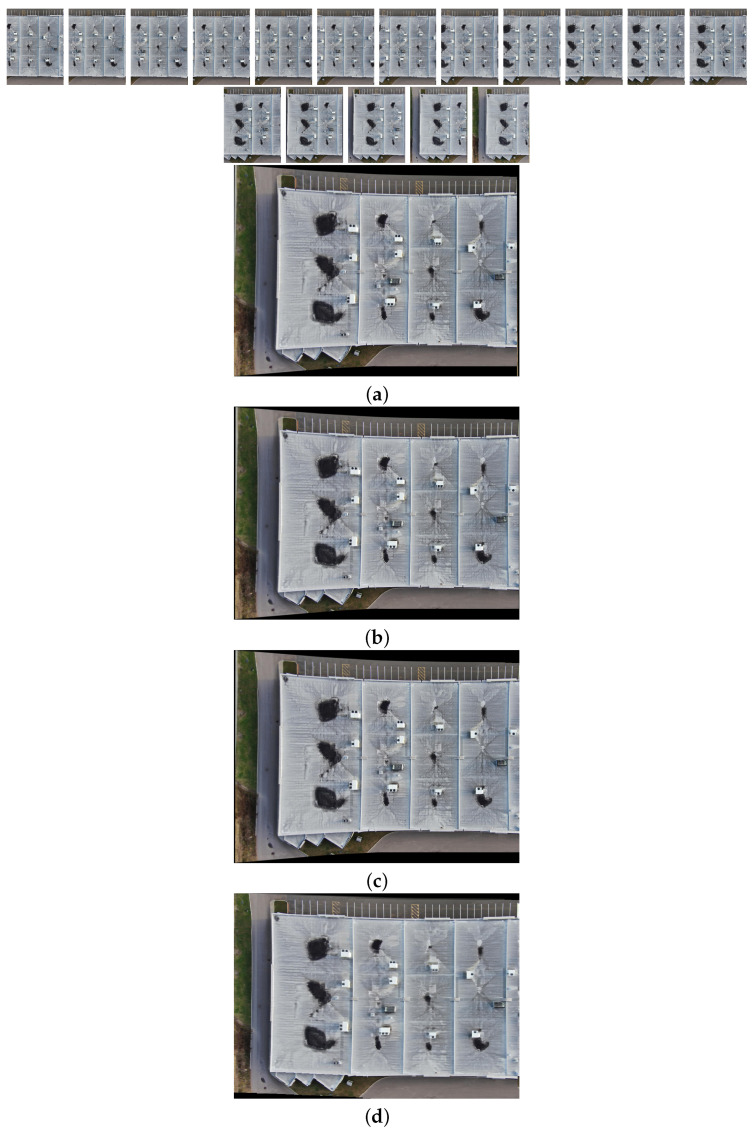
Image stitching for seventeen images of dataset 1. (**a**) AKAZE + BFMatcher: the stitched image has perspective distortion; (**b**) ORB + BFMatcher: the stitched image has perspective distortion; (**c**) SIFT + BFMatcher: the stitched image has perspective distortion; (**d**) proposed method: the stitched image is regular and perspective distortion free.

**Figure 18:**
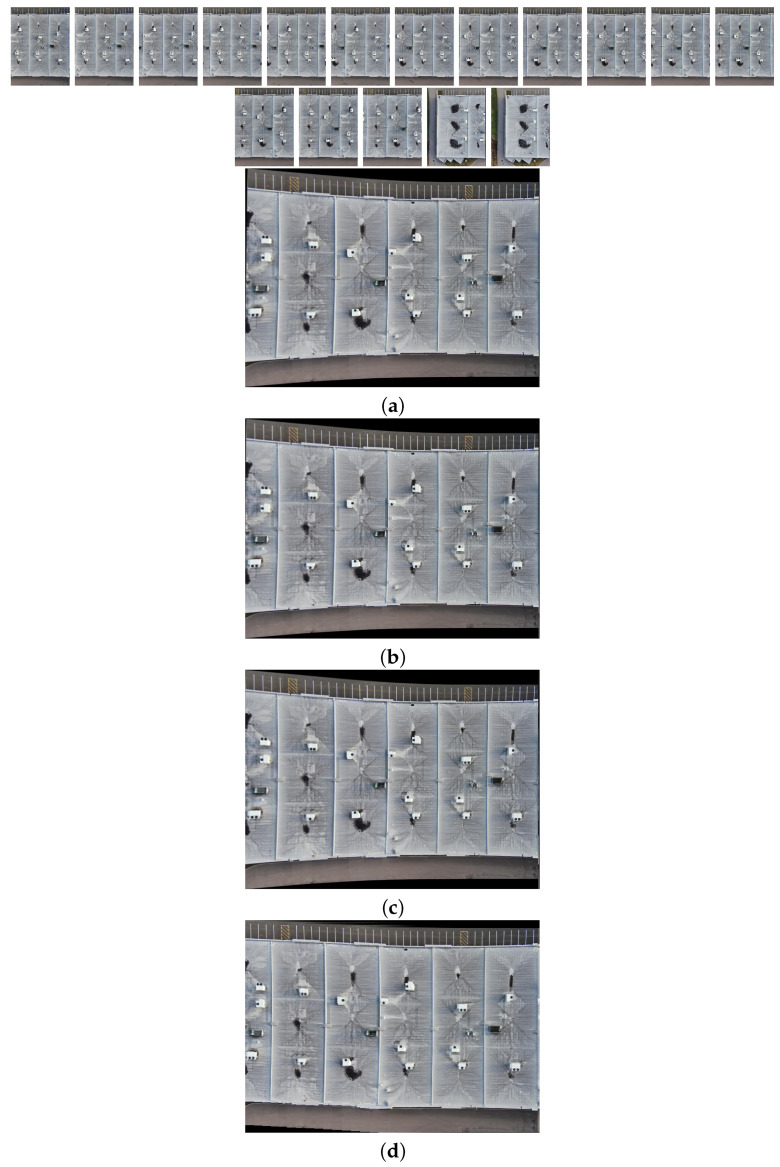
Image stitching for seventeen images of dataset 1. (**a**) AKAZE + BFMatcher: the stitched image has perspective distortion; (**b**) ORB + BFMatcher: the stitched image has perspective distortion; (**c**) SIFT + BFMatcher: the stitched image has perspective distortion; (**d**) proposed method: the stitched image is regular and perspective distortion free.

**Figure 19:**
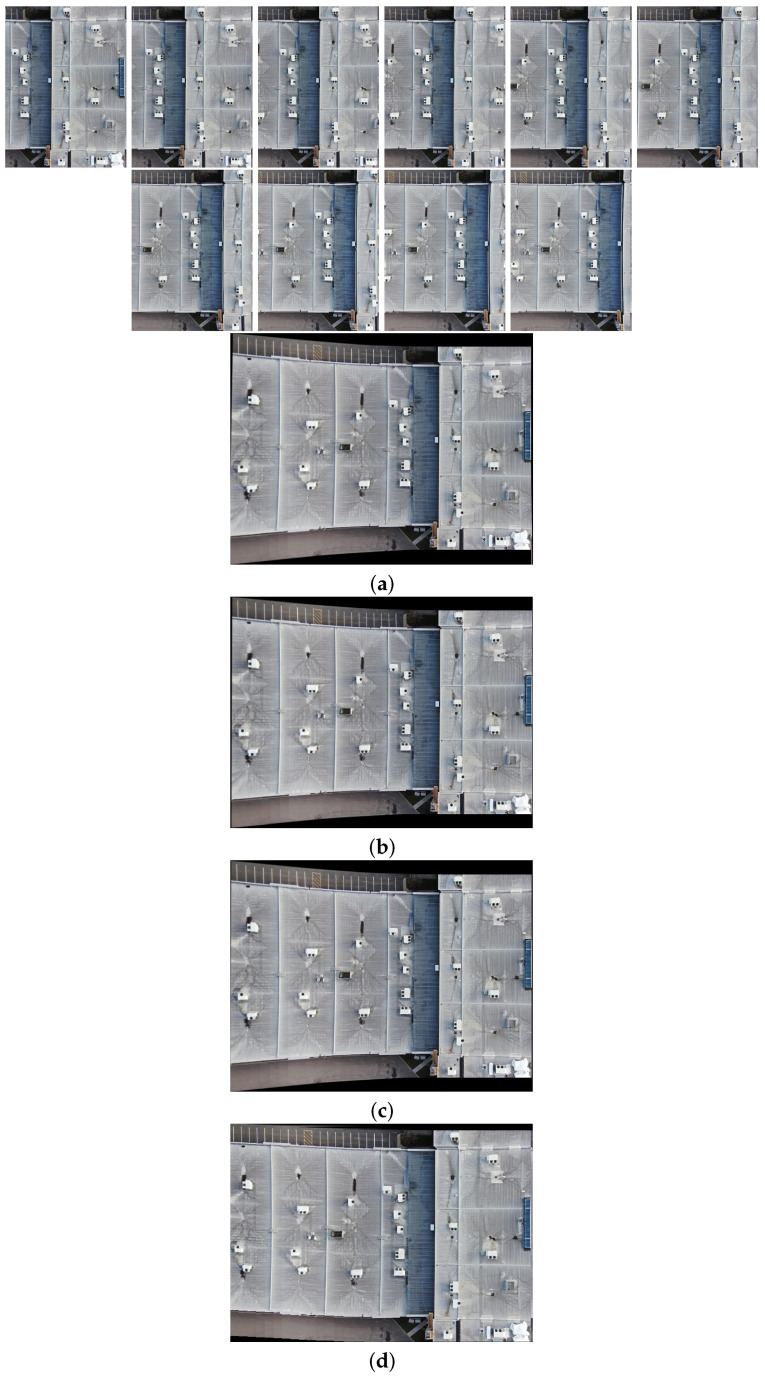
Image stitching for ten images of dataset 1. (**a**) AKAZE + BFMatcher: the stitched image has perspective distortion; (**b**) ORB + BFMatcher: the stitched image has perspective distortion; (**c**) SIFT + BFMatcher: the stitched image has perspective distortion; (**d**) proposed method: the stitched image is regular and perspective distortion free.

**Figure 20:**
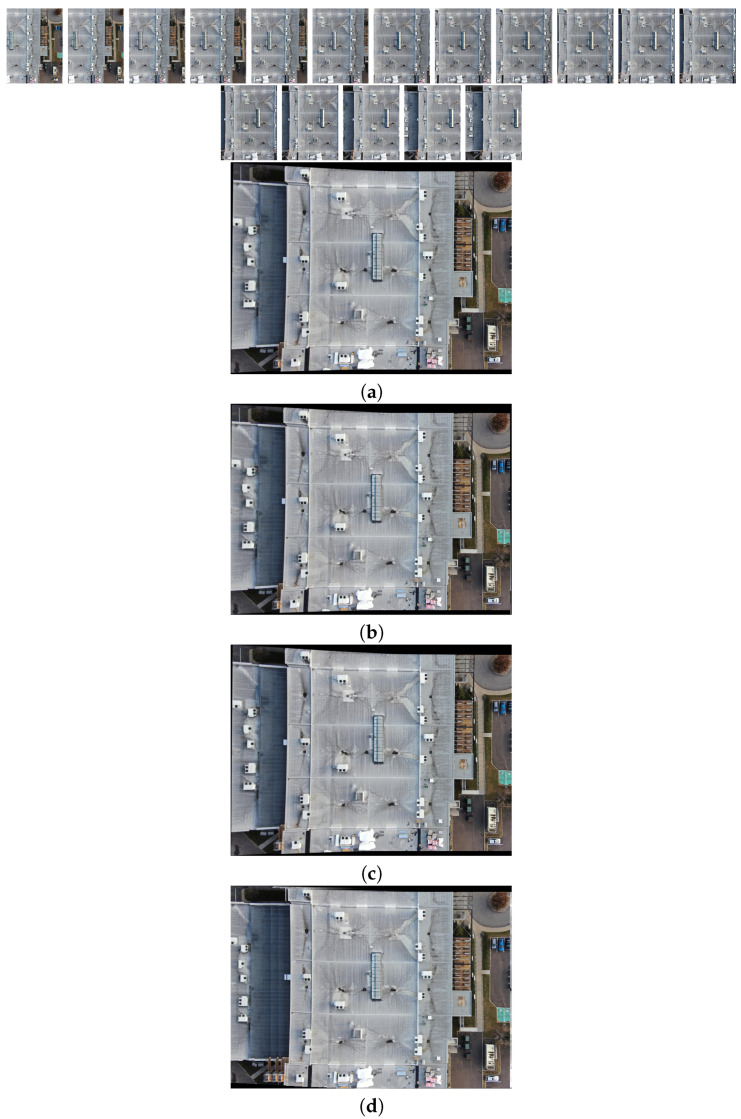
Image stitching for seventeen images of dataset 1. (**a**) AKAZE + BFMatcher: the stitched image has perspective distortion; (**b**) ORB + BFMatcher: the stitched image has perspective distortion; (**c**) SIFT + BFMatcher: the stitched image has perspective distortion; (**d**) proposed method: the stitched image is regular and perspective distortion free.

**Figure 21:**
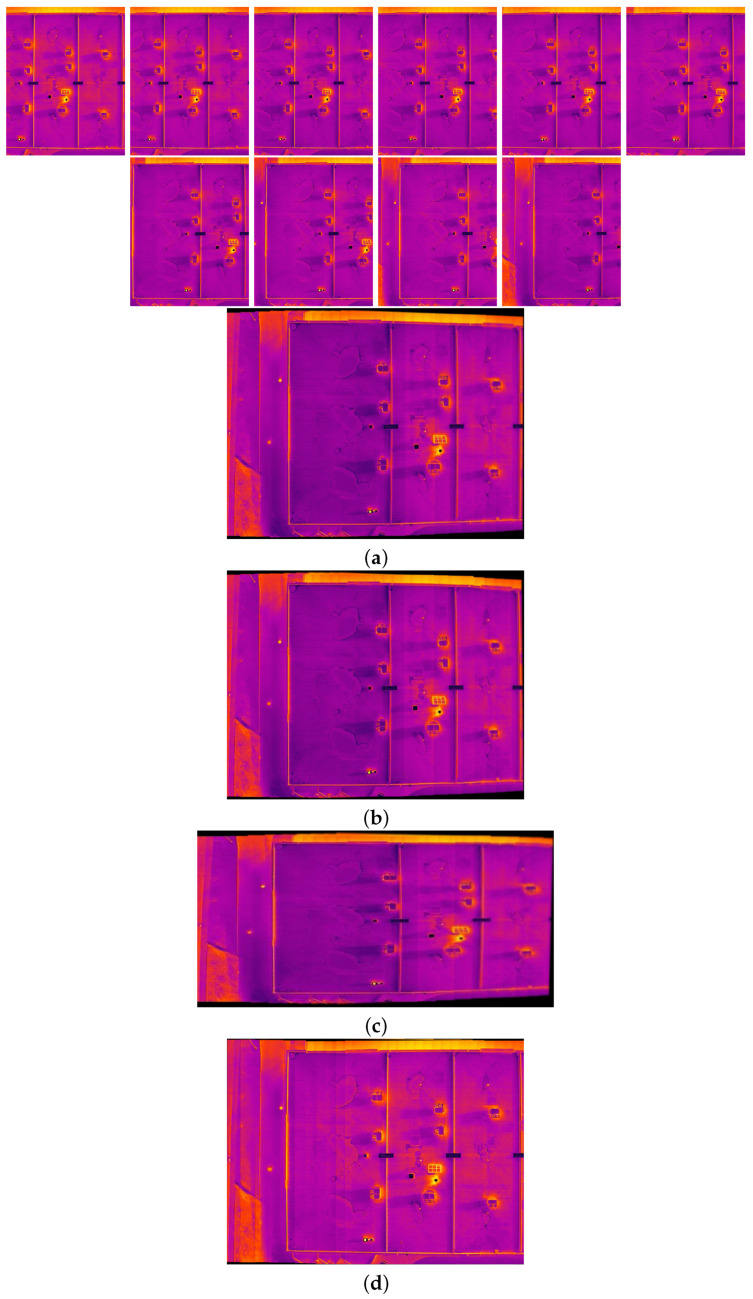
Image stitching for ten images of dataset 1. (**a**) AKAZE + BFMatcher: the stitched image has perspective distortion; (**b**) ORB + BFMatcher: the stitched image has perspective distortion; (**c**) SIFT + BFMatcher: the stitched image has perspective distortion; (**d**) proposed method: the stitched image is regular and perspective distortion free.

**Figure 22:**
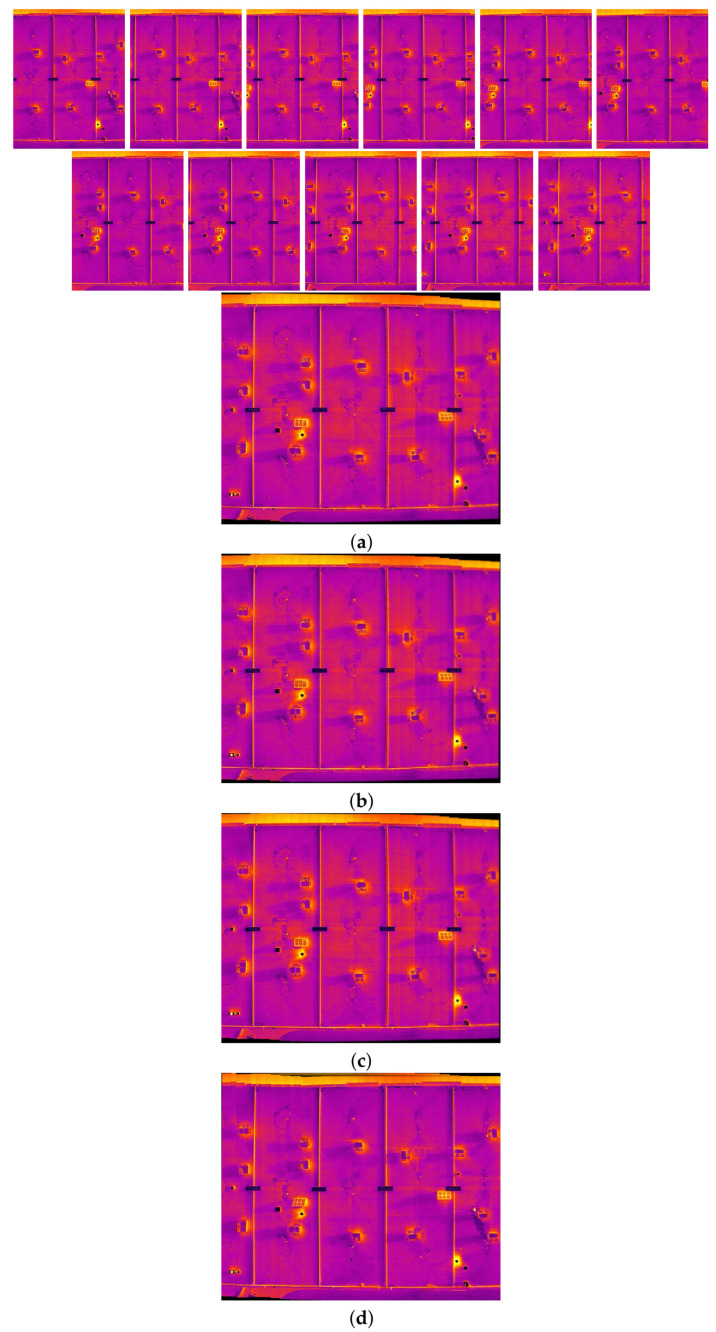
Image stitching for eleven images of dataset 1. (**a**) AKAZE + BFMatcher: the stitched image has perspective distortion; (**b**) ORB + BFMatcher: the stitched image has perspective distortion; (**c**) SIFT + BFMatcher: the stitched image has perspective distortion; (**d**) proposed method: the stitched image is regular and perspective distortion free.

**Figure 23:**
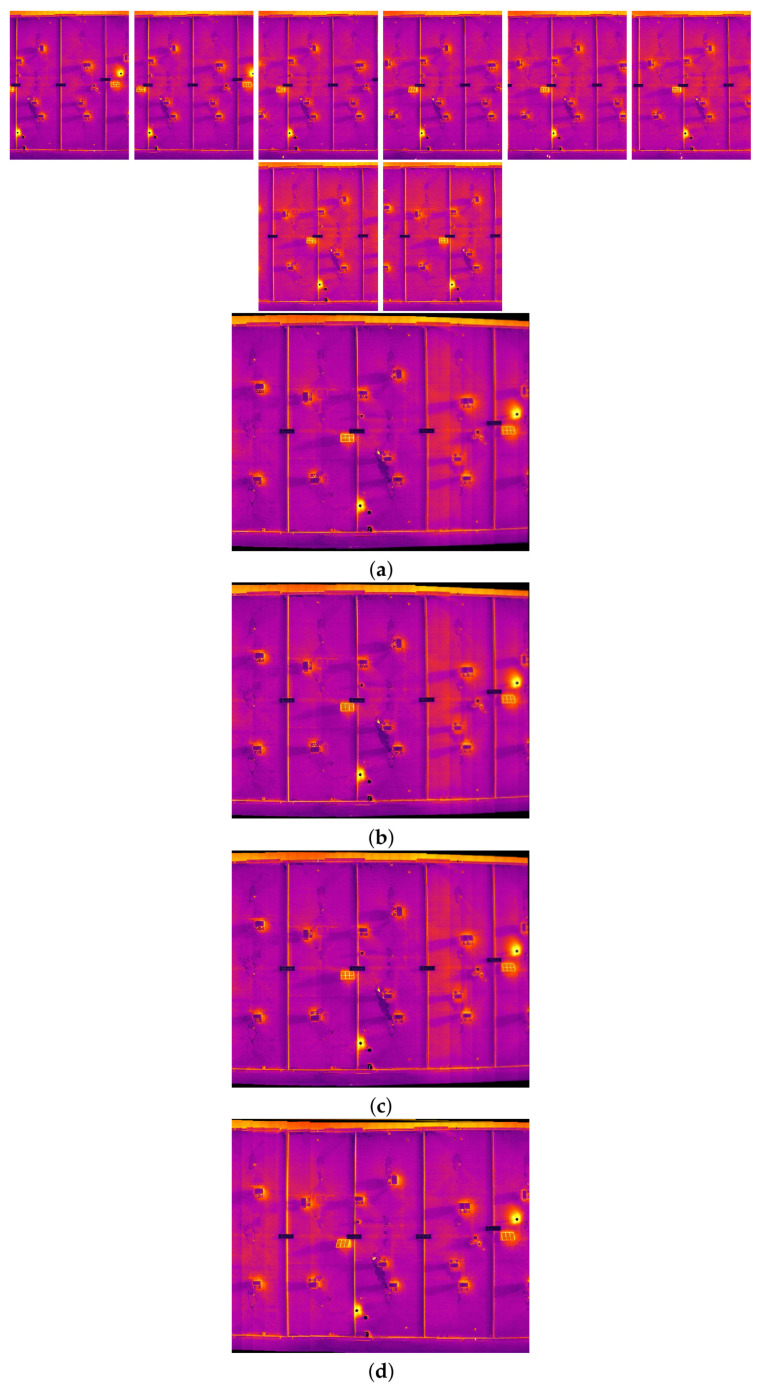
Image stitching for eight images of dataset 1. (**a**) AKAZE + BFMatcher: the stitched image has perspective distortion; (**b**) ORB + BFMatcher: the stitched image has perspective distortion; (**c**) SIFT + BFMatcher: the stitched image has perspective distortion; (**d**) proposed method: the stitched image is regular and perspective distortion free.

**Figure 24:**
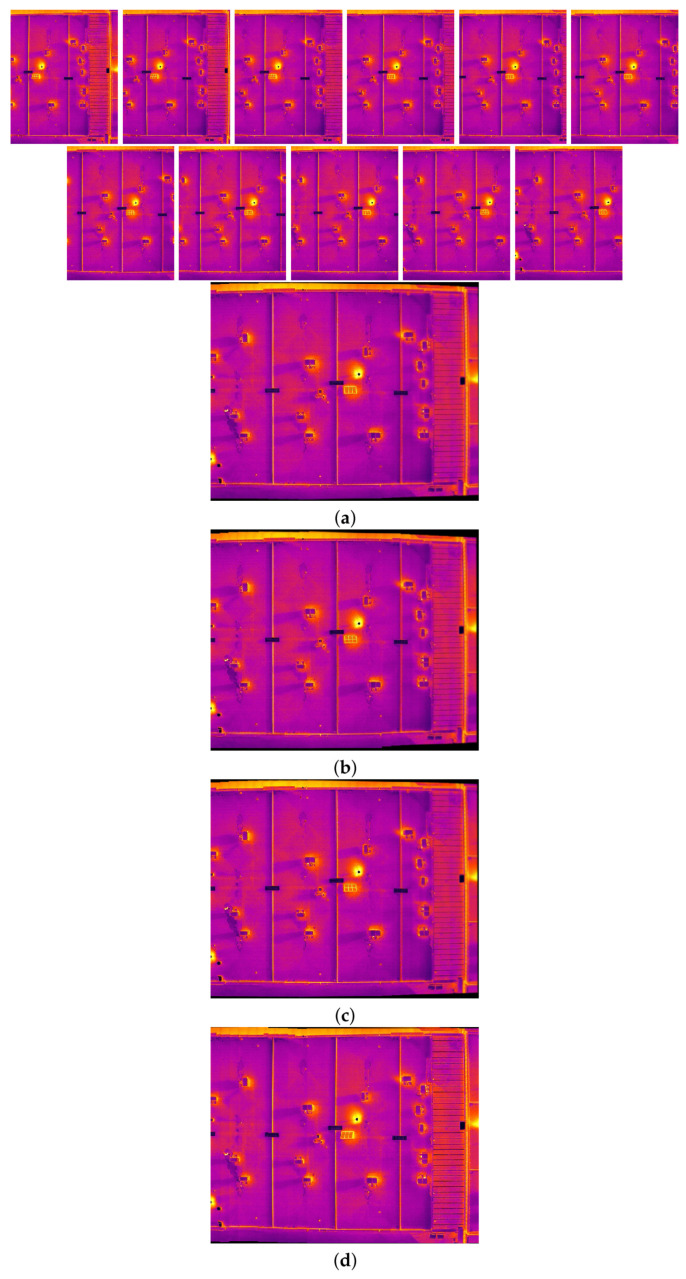
Image stitching for eleven images of dataset 1. (**a**) AKAZE + BFMatcher: the stitched image has perspective distortion; (**b**) ORB + BFMatcher: the stitched image has perspective distortion; (**c**) SIFT + BFMatcher: the stitched image has perspective distortion; (**d**) proposed method: the stitched image is regular and perspective distortion free.

**Figure 25:**
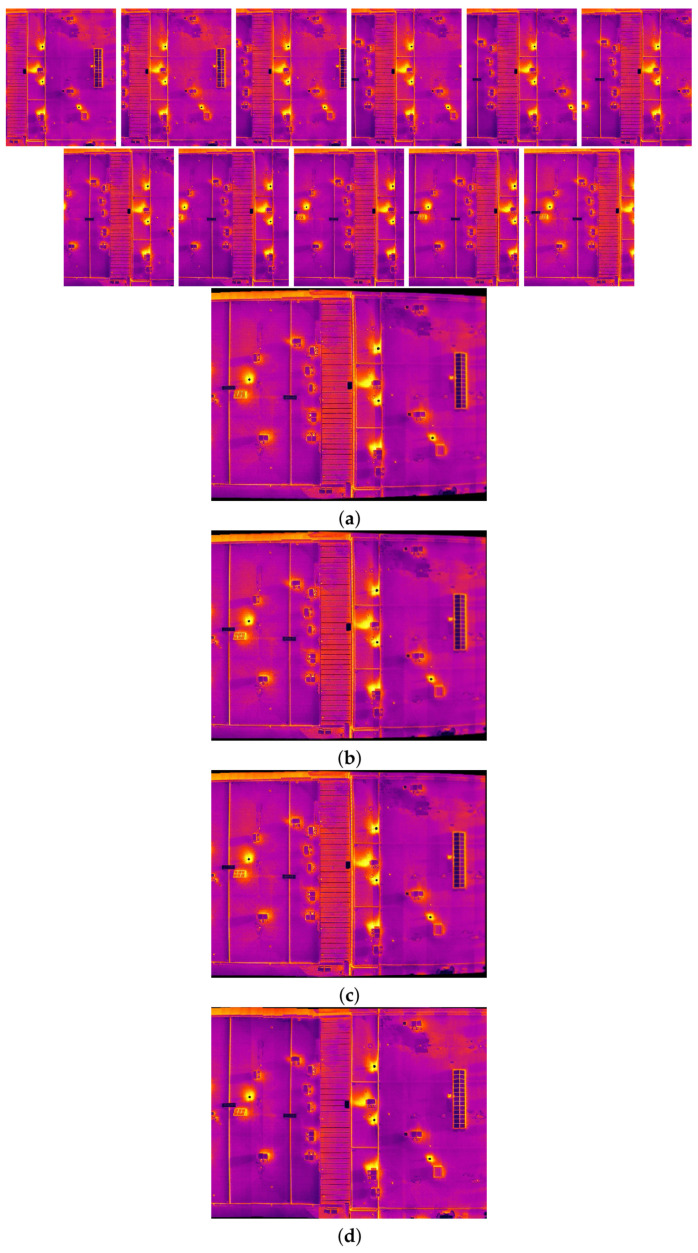
Image stitching for eleven images of dataset 1. (**a**) AKAZE + BFMatcher: the stitched image has perspective distortion; (**b**) ORB + BFMatcher: the stitched image has perspective distortion; (**c**) SIFT + BFMatcher: the stitched image has perspective distortion; (**d**) proposed method: the stitched image is regular and perspective distortion free.

**Figure 26:**
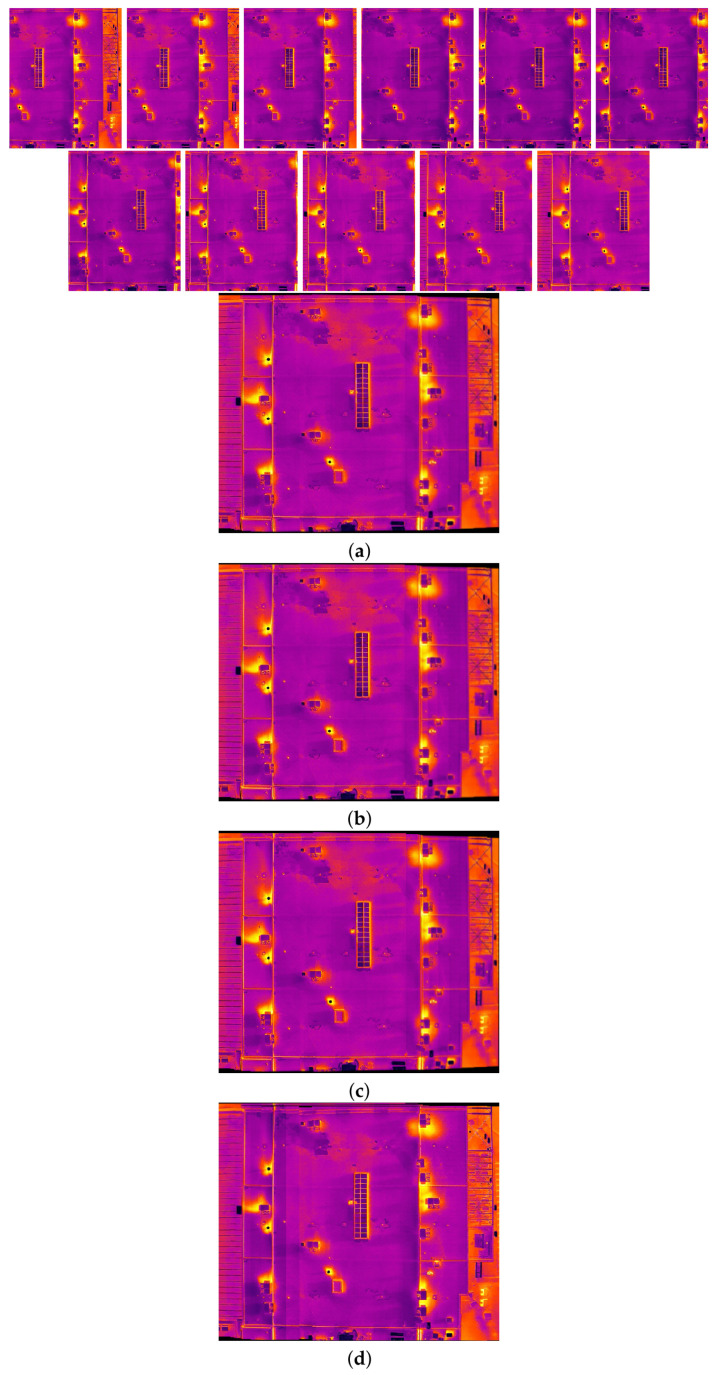
Image stitching for eleven images of dataset 1. (**a**) AKAZE + BFMatcher: the stitched image has perspective distortion; (**b**) ORB + BFMatcher: the stitched image has perspective distortion; (**c**) SIFT + BFMatcher: the stitched image has perspective distortion; (**d**) proposed method: the stitched image is regular and perspective distortion free.

**Figure 27:**
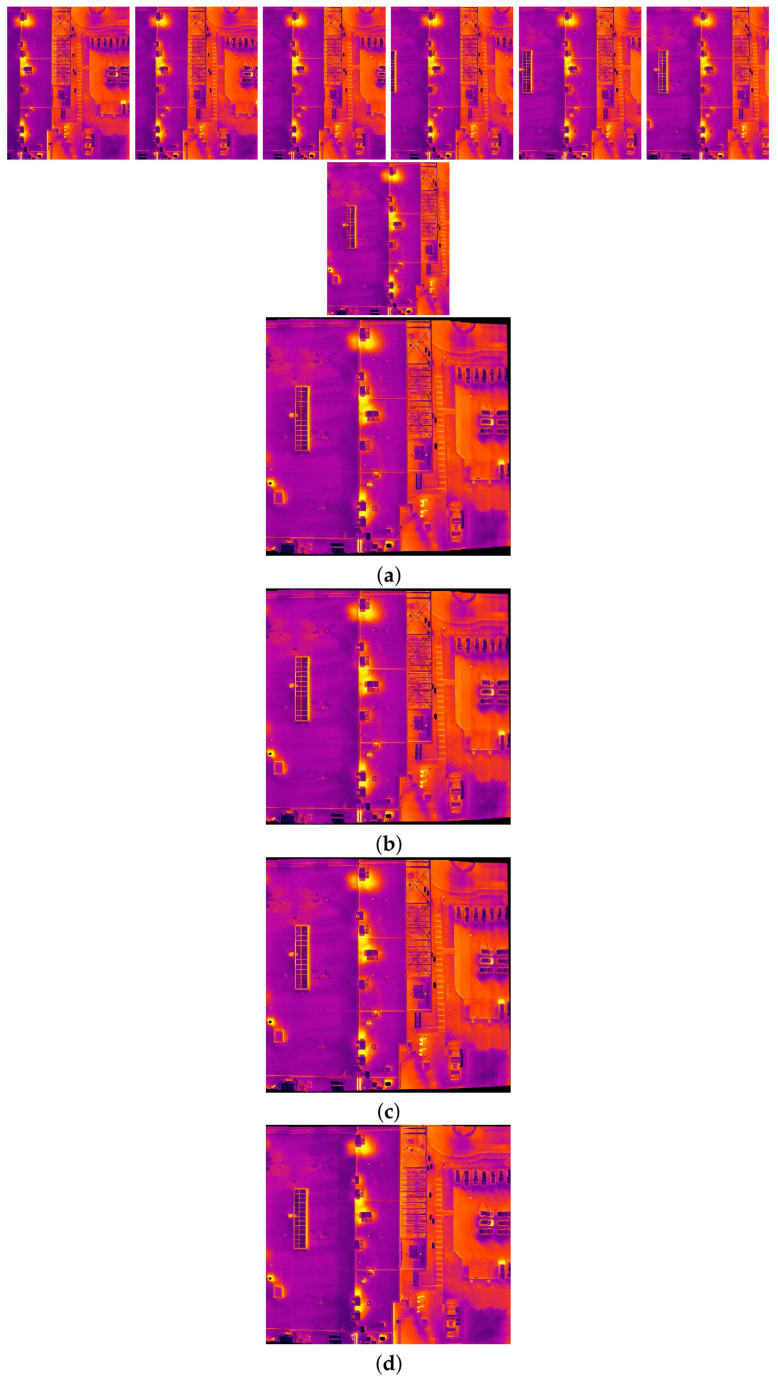
Image stitching for seven images of dataset 1. (**a**) AKAZE + BFMatcher: the stitched image has perspective distortion; (**b**) ORB + BFMatcher: the stitched image has perspective distortion; (**c**) SIFT + BFMatcher: the stitched image has perspective distortion; (**d**) proposed method: the stitched image is regular and perspective distortion free.

Figure 17, Figure 18, Figure 19 and Figure 20 show four scenarios of the visible images of dataset 1 and the final results of the proposed image stitching method. As the figures indicate, the proposed method brings a panorama perspective-distortion-free image in comparison to the other methods. Figure 21, Figure 22, Figure 23, Figure 24, Figure 25, Figure 26 and Figure 27 indicate seven scenarios of the counterpart infrared images and the final results of the proposed image stitching method. As the figures indicate, the proposed method brings out a panorama perspective-distortion-free image in comparison to the other methods.

## 5. Conclusions

Efficient image stitching emerges as an essential component in advancing the Non-Destructive Evaluation (NDE) of infrastructures, addressing the imperative need for precise defect visualization within large structures. The conventional reliance on high-resolution close-distance images for defect detection encounters challenges in comprehensively understanding the location and continuity of defects, particularly those minor defects which may not be captured from a significant distance. To address this, the proposed image stitching method for dual-sensor inspections not only offers enhanced defect visualization but also accommodates automated inspection for structural maintenance.

The significance of this work lies in recognizing the limitations of existing methodologies and addressing the pronounced demand for an effective image stitching technique appropriate to infrared and visible images within complex structures and industrial assets. In response to this need, we proposed a unified self-supervised auto-encoder feature detection to augment the quality and quantity of feature detection. Then, utilizing a graph neural network for robust feature matching, our technique surpassed existing approaches in eliminating perspective distortion in both infrared and visible images. This distortion correction is a critical prerequisite for subsequent multi-modal fusion strategies for NDE of infrastructures.

The results of the proposed method substantially improve the visualization capabilities for infrastructure inspection. Through comparative analyses with state-of-the-art methods, the proposed approach consistently demonstrated superiority, underscoring its efficacy in addressing the unique challenges posed by dual-sensor inspections. Rigorous comparative evaluations against established techniques further validated the effectiveness of the proposed approach, emphasizing its potential to redefine the landscape of image stitching in the realm of infrastructure inspection.

In conclusion, our advanced image stitching method stands poised as a transformative contribution to the field, offering a powerful tool for defect detection and visualization across extensive structures. The demonstrated efficacy of the approach paves the way for enhanced inspection methodologies, underscoring the potential for broader applications in the realm of Non-Destructive Evaluation and structural maintenance.

For future work, our work lays the groundwork for continued advancements in image stitching technologies, promising a future where the seamless fusion of infrared and visible imaging will become a cornerstone in infrastructure assessment and maintenance practices and assist profoundly in improving the non-destructive testing industry.

## Data Availability

Data are contained within the article.

## Abbreviations

The following abbreviations are used in this manuscript:

IRT	Infrared thermography
NDE	Non-Destructive Evaluation
NDT	Non-destructive testing
CNN	Convolutional neural network
AKAZE	Accelerated Keypoint and Zernike Moment Descriptor
ORB	Oriented FAST and Rotated BRIEF
SIFT	Scale-Invariant Feature Transform
RANSAC	Random Sample Consensus

## References

[1] Kot P., Muradov M., Gkantou M., Kamaris G.S., Hashim K., Yeboah D. (2021). Recent advancements in non-destructive testing techniques for structural health monitoring. Appl. Sci..

[2] Fuentes S., Tongson E., Gonzalez Viejo C. (2021). Urban green infrastructure monitoring using remote sensing from integrated visible and thermal infrared cameras mounted on a moving vehicle. Sensors.

[3] Chen J., Li Z., Peng C., Wang Y., Gong W. (2022). UAV Image Stitching Based on Optimal Seam and Half-Projective Warp. Remote Sens..

[4] Shahsavarani S., Lopez F., Maldague X. (2023). Multi-modal image processing pipeline for NDE of structures and industrial assets. Thermosense: Thermal Infrared Applications XLV.

[5] Ghiass R.S., Arandjelović O., Bendada H., Maldague X. (2014). A unified framework for thermal face recognition. Neural Information Processing, Proceedings of the 21st International Conference, ICONIP 2014, Kuching, Malaysia, 3–6 November 2014.

[6] Ghiass R.S., Arandjelović O., Bendada H., Maldague X. Illumination-invariant face recognition from a single image across extreme pose using a dual dimension AAM ensemble in the thermal infrared spectrum. Proceedings of the 2013 International Joint Conference on Neural Networks (IJCNN).

[7] Nooralishahi P., Ramos G., Pozzer S., Ibarra-Castanedo C., Lopez F., Maldague X.P. (2022). Texture analysis to enhance drone-based multi-modal inspection of structures. Drones.

[8] Yi K.M., Trulls E., Lepetit V., Fua P. Lift: Learned invariant feature transform. Proceedings of the European Conference on Computer Vision.

[9] Zhang L., Rusinkiewicz S. Learning to detect features in texture images. Proceedings of the IEEE Conference on Computer Vision and Pattern Recognition.

[10] DeTone D., Malisiewicz T., Rabinovich A. Superpoint: Self-supervised interest point detection and description. Proceedings of the IEEE Conference on Computer Vision and Pattern Recognition Workshops.

[11] Lenc K., Vedaldi A. Learning covariant feature detectors. Proceedings of the European Conference on Computer Vision.

[12] Savinov N., Seki A., Ladicky L., Sattler T., Pollefeys M. Quad-networks: Unsupervised learning to rank for interest point detection. Proceedings of the IEEE Conference on Computer Vision and Pattern Recognition.

[13] Ono Y., Trulls E., Fua P., Yi K.M. LF-NET: Learning local features from images. Proceedings of the Advances in Neural Information Processing Systems.

[14] Georgakis G., Karanam S., Wu Z., Ernst J., Kosecká J. End-to-end learning of keypoint detector and descriptor for pose invariant 3D matching. Proceedings of the IEEE Conference on Computer Vision and Pattern Recognition.

[15] Barroso-Laguna A., Riba E., Ponsa D., Mikolajczyk K. Key.net: Keypoint detection by handcrafted and learned CNN filters. Proceedings of the IEEE International Conference on Computer Vision.

[16] Verdie Y., Yi K., Fua P., Lepetit V. Tilde: A temporally invariant learned detector. Proceedings of the IEEE Conference on Computer Vision and Pattern Recognition.

[17] Zitova B., Flusser J. (2003). Image registration methods: A survey. Image Vis. Comput..

[18] Litjens G., Kooi T., Bejnordi B.E., Setio A.A.A., Ciompi F., Ghafoorian M. (2017). A survey on deep learning in medical image analysis. Med. Image Anal..

[19] Dawn S., Saxena V., Sharma B. Remote sensing image registration techniques: A survey. Proceedings of the International Conference on Image and Signal Processing.

[20] Sotiras A., Davatzikos C., Paragios N. (2013). Deformable medical image registration: A survey. IEEE Trans. Med. Imaging.

[21] Ferrante E., Paragios N. (2017). Slice-to-volume medical image registration: A survey. Med. Image Anal..

[22] Lawler E.L. (1963). The quadratic assignment problem. Manag. Sci..

[23] Belongie S., Malik J., Puzicha J. Shape context: A new descriptor for shape matching and object recognition. Proceedings of the Advances in Neural Information Processing Systems.

[24] Loiola E.M., de Abreu N.M.M., Boaventura-Netto P.O., Hahn P., Querido T. (2007). A survey for the quadratic assignment problem. Eur. J. Oper. Res..

[25] Ghosh D., Kaabouch N., Hu W.C. (2016). A robust iterative super-resolution mosaicking algorithm using an adaptive and directional Huber-Markov regularization. J. Vis. Commun. Image Represent..

[26] Brown M., Lowe D.G. (2007). Automatic panoramic image stitching using invariant features. Int. J. Comput. Vis..

[27] Lowe D. (2004). Distinctive Image Features from Scale-Invariant Keypoints. Int. J. Comput. Vis..

[28] Fischler M.A., Bolles R.C. (1981). Random sample consensus: A paradigm for model fitting with applications to image analysis and automated cartography. Commun. ACM.

[29] Lin W.Y., Liu S., Matsushita Y., Ng T.T., Cheong L.F. Smoothly varying affine stitching. Proceedings of the IEEE Conference on Computer Vision and Pattern Recognition.

[30] Hafeez J., Lee J., Kwon S., Ha S., Hur G., Lee S. (2020). Evaluating feature extraction methods with synthetic noise patterns for image-based modelling of texture-less objects. Remote Sens..

[31] Sun J., Shen Z., Wang Y., Bao H., Zhou X. LoFTR: Detector-free local feature matching with transformers. Proceedings of the IEEE/CVF Conference on Computer Vision and Pattern Recognition.

[32] Wang Y., Yao H., Zhao S. (2016). Auto-encoder based dimensionality reduction. Neurocomputing.

[33] Tschannen M., Bachem O., Lucic M. (2018). Recent advances in autoencoder-based representation learning. arXiv.

[34] Simonyan K., Zisserman A. (2014). Very deep convolutional networks for large-scale image recognition. arXiv.

[35] Liu W., Wen Y., Yu Z., Yang M. Large-Margin Softmax Loss for Convolutional Neural Networks. Proceedings of the International Conference on Machine Learning.

[36] Neubeck A., Van Gool L. Efficient Non-Maximum Suppression. Proceedings of the 18th International Conference on Pattern Recognition (ICPR’06).

[37] Cao S.Y., Hu J., Sheng Z., Shen H.L. Iterative Deep Homography Estimation. Proceedings of the IEEE/CVF Conference on Computer Vision and Pattern Recognition.

[38] Lin T.Y., Maire M., Belongie S., Hays J., Perona P., Ramanan D., Zitnick C.L. (2014). Microsoft COCO: Common Objects in Context. Proceedings of the Computer Vision—ECCV 2014: 13th European Conference.

[39] Sarlin P.E., DeTone D., Malisiewicz T., Rabinovich A. Superglue: Learning feature matching with graph neural networks. Proceedings of the IEEE/CVF Conference on Computer Vision and Pattern Recognition.

[40] Rublee E., Rabaud V., Konolige K., Bradski G. ORB: An efficient alternative to SIFT or SURF. Proceedings of the 2011 International Conference on Computer Vision.

[41] Alcantarilla P.F., Bartoli A., Davison A.J. KAZE features. Proceedings of the Computer Vision—ECCV 2012: 12th European Conference on Computer Vision.

